# A Novel Method for Gene-Specific Enhancement of Protein Translation by Targeting 5’UTRs of Selected Tumor Suppressors

**DOI:** 10.1371/journal.pone.0155359

**Published:** 2016-05-12

**Authors:** Adam Master, Anna Wójcicka, Kamilla Giżewska, Piotr Popławski, Graham R. Williams, Alicja Nauman

**Affiliations:** 1 The Centre of Postgraduate Medical Education, Department of Biochemistry and Molecular Biology, ul. Marymoncka 99/103, 01-813, Warsaw, Poland; 2 BioTe21, Laboratory of Molecular Medical Biology, ul. Krolowej Jadwigi 33/3b, 30-209, Cracow, Poland; 3 Centre of New Technologies, University of Warsaw, Banacha 2c, 02-089, Warsaw, Poland; 4 Genomic Medicine, Medical University of Warsaw, Zwirki i Wigury 61, 02-091, Warsaw, Poland; 5 Molecular Endocrinology Group, Department of Medicine, Imperial College London, Hammersmith Campus, London, W12 0NN, United Kingdom; University of Surrey, UNITED KINGDOM

## Abstract

**Background:**

Translational control is a mechanism of protein synthesis regulation emerging as an important target for new therapeutics. Naturally occurring microRNAs and synthetic small inhibitory RNAs (siRNAs) are the most recognized regulatory molecules acting via RNA interference. Surprisingly, recent studies have shown that interfering RNAs may also activate gene transcription via the newly discovered phenomenon of small RNA-induced gene activation (RNAa). Thus far, the small activating RNAs (saRNAs) have only been demonstrated as promoter-specific transcriptional activators.

**Findings:**

We demonstrate that oligonucleotide-based *trans*-acting factors can also specifically enhance gene expression at the level of protein translation by acting at sequence-specific targets within the messenger RNA 5’-untranslated region (5’UTR). We designed a set of short synthetic oligonucleotides (dGoligos), specifically targeting alternatively spliced 5’UTRs in transcripts expressed from the *THRB* and *CDKN2A* suppressor genes. The *in vitro* translation efficiency of reporter constructs containing alternative TRβ1 5’UTRs was increased by up to more than 55-fold following exposure to specific dGoligos. Moreover, we found that the most folded 5’UTR has higher translational regulatory potential when compared to the weakly folded TRβ1 variant. This suggests such a strategy may be especially applied to enhance translation from relatively inactive transcripts containing long 5’UTRs of complex structure.

**Significance:**

This report represents the first method for gene-specific translation enhancement using selective *trans*-acting factors designed to target specific 5’UTR *cis*-acting elements. This simple strategy may be developed further to complement other available methods for gene expression regulation including gene silencing. The dGoligo-mediated translation-enhancing approach has the potential to be transferred to increase the translation efficiency of any suitable target gene and may have future application in gene therapy strategies to enhance expression of proteins including tumor suppressors.

## Introduction

Translational control is one of the most important mechanisms of post-transcriptional regulation of gene expression, determining final protein levels [1]. Initiation of translation [2] is a rate-limiting phase of protein synthesis, controlled by translation -silencing or -enhancing *cis*-acting elements located in the 5’ and 3’ untranslated regions (5’UTR, 3’UTR) of mRNAs [3]. The best studied *cis*-acting elements within the UTRs are the upstream open reading frames (uORFs) [4] and internal ribosomal entry sites (IRESs) [5]. These regulatory sequences may be organized in secondary and tertiary RNA structures that are recognized by *trans*-acting factors [6] such as translation factors [7], naturally occurring microRNAs (microRNAs) [8] as well as synthetic small interfering RNAs (siRNAs) [9] and antisense-like oligonucleotides (ASOs) usually lowering final protein levels [10].

Translation of most human mRNAs occurs via a 5’-cap-dependent mechanism [11]. Certain physiological or pathological factors may switch the classic mechanism to an alternative one that can be controlled by an mRNA element such as uORF, IRES, iron responsive element (IRE), RNA hypoxia response element (rHRE), differentiation-control element (DICE) or cap-independent translational enhancer (CITE) [12, 13]. An alternate cap-independent IRES-dependent translation [5, 13] is activated in some cellular phases such as cell division [14] and during integrated stress responses (ISR) [15] caused by heat shock, serum or amino-acid deprivation and in hypoxia, as frequently observed in solid tumors [16]. Expression of specific genes involved in the stress responses can be also controlled by uORFs [4, 13]. ISR-enhanced synthesis of ATF4 (Activating Transcription Factor 4) protein is an extensively-studied model of the translational control [4]. This mechanism involves the differential contribution of two uORFs: the 5’ proximal uORF1 that is a positive *cis*-acting element and the inhibitory uORF2 overlapping correct ATF4 ORF in an out of frame manner. Non-stressed, normal conditions allow cells for fast translation of the short uORF1 and ribosome reinitiation at the uORF2, that results in synthesis of truncated proteins. In contrast, stress conditions increase the time required for more accurate scanning that allows the ribosomes to bypass the inhibitory uORF2 and reinitiate at the downstream ATF4-coding region [4]. Translation initiation can also be slowed down by various interfering *trans*-acting factors [1] or highly-ordered RNA structures [17], which require RNA helicases to be scanned through [3, 13]. Moreover, 5’UTR structures, recruiting RNA helicase eIF4A2, have now been demonstrated to play a crucial role in 3’UTR-dependent, microRNA-mediated gene silencing [18]. Therefore, efficiency of various mechanisms involved in translation initiation has been thought to be dependent on the folding state of 5’UTRs, determined by the Gibbs energy (ΔG) [17].

Many genes have several alternative 5’UTR splice variants that can differentially regulate translation of downstream coding sequences [6]. One example of such a complex gene is *THRB* (GeneID 7068), which encodes β isoforms of human thyroid hormone receptors (TRβ1, TRβ2 and TRβ4) [19] and contains numerous alternatively spliced exons that generate various alternate 5’UTRs in mRNAs from which the TRβ tumor suppressor protein is expressed [19, 20]. Multiple strongly folded 5’UTRs can also be expressed by another tumor suppressor–*CDKN2A* (GeneID 1029) [21]. Both genes encode 5’UTRs containing numerous uORFs [21, 22] and IRES-like sequences [21, 23]. These 5’UTRs vary in length, GC-content and secondary structure and have been shown to influence the efficiency of protein translation [21, 23].

Recent studies have revealed that some naturally occurring microRNAs, considered traditionally as inhibitory *trans*-acting factors that bind to 3’UTR sequences, can also up-regulate protein synthesis [24]. Moreover, it has been discovered that several mRNAs contain similar microRNA targets termed miBridges present in both 3'UTR and 5'UTR regions that can bind the same microRNA molecule [25]. Further supporting the hypothesis of microRNA binding to 5’UTRs, a liver-specific microRNA, miR-122 has been shown to stimulate synthesis of hepatitis C virus (HCV) protein by direct interaction with two target sites in the 5’UTR of the HCV genome [26]. Even though a single microRNA usually targets possibly hundreds of cellular mRNAs [27], showing low selectivity towards transcripts of a single gene [28], these findings demonstrate a new role of short interfering RNAs that may lead not only to gene repression, but also to protein synthesis enhancement.

Recently, a new type of RNA interference has been shown to result from promoter-specific activation of gene transcription (RNAa, RNA activation) that is triggered by a novel class of interfering RNAs termed small activating RNAs (saRNAs), which target discrete promoter sequences [29]. The saRNAs were used for promoter-specific upregulation of gene transcription [30]. On the other hand, the saRNAs were alternatively reported to represent siRNAs that bind to and inhibit long naturally occurring antisense transcripts (NATs) overlapping complementary promoter regions of target genes, which play a role in transcriptional repression [31, 32]. Thus, silencing of the NATs could indirectly lead to transcriptional activation of the genes [33, 34]. Both mechanisms of gene regulation, however, have been shown to control only the levels of mRNA expression [31, 33].

Here we describe a novel method for 5’UTR-specific enhancement of translation. Protein overexpression is triggered by synthetic, translation-enhancing oligonucleotides, termed dGoligos (dGs), which were originally designed to alter Gibbs energy-dependent secondary structure formation of specific sequences of TRβ1 5’UTRs. Although ΔG is a well-known measure of the stability of higher-order structures of nucleic acid molecules, we used this parameter in a new way, defined in a bespoke dGenhancer calculator. This tool allowed us to determine *cis*-acting elements within TRβ1 5’UTRs that were recognized by dGs. Then, the translation-enhancing effects were successfully confirmed by the use of dGs design to target p16^INK4a^ 5’UTR encoded by the *CDKN2A* gene. dGoligos thus offer the potential for a novel and specific gene-therapy approach to re-express or over-express individual proteins such as tumor suppressors.

## Results

### Translation regulated by differentially folded TRβ1 5’UTRs

**Fig 1:**
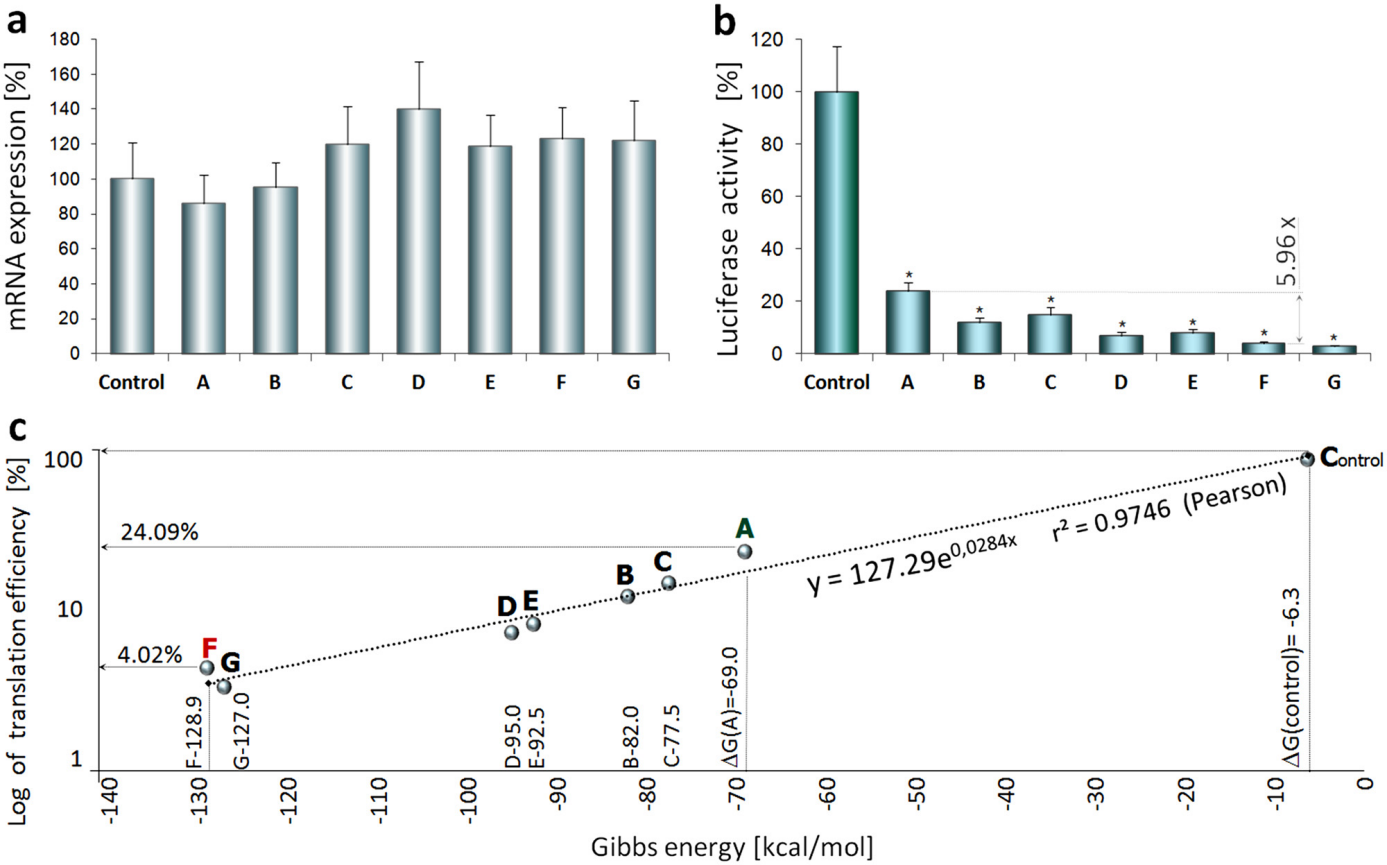
Correlation between Gibbs energy and basic TRβ1 5'UTR-mediated translation efficiency. (**a**) Luciferase mRNA levels from *in vitro* wheat germ-based coupled transcription-translation assay performed on plasmids containing TRβ1 5’UTR variants A-G are shown relative to control plasmid. (**b**) Effects of 5’UTR variants A-G on luciferase activities after 6h coupled transcription-translation. Three independent experiments were performed in triplicate and shown as mean % mRNA or luciferase activity± SD. Data were analyzed by ANOVA followed by Dunnett’s multiple comparison test; *p< 0.001 vs. control. **c**, Correlation between the calculated Gibbs energies (X axis) of each 5’UTR variant (S1 Table) and UTR-mediated luciferase translation efficiency. The correlation is shown as the exponential trend-line y = 127.29_*_e^0,0248*X^, where x = calculated Gibbs energy; Pearson product-moment correlation coefficient r² = 0,9746. Logarithmically transformed data of translation efficiency (Y axis) were analyzed together with Gibbs energies by linear regression; p = 0.0123.

TRβ1 5’UTR splice variants A-G subcloned upstream of the luciferase reporter gene in pKS plasmids [22] were tested for their basic expression characteristics by coupled *in vitro* transcription-translation (RTS 100 Wheat Germ CECF). The basic luciferase levels served as starting points for further studies on translation-enhancing elements of the 5’UTRs. Initial results demonstrated that variants A-G differently regulate the reporter protein translation efficiency (Fig 1a and 1b). The measurements were shown in relation to control plasmid (pKS-control) containing an irrelevant synthetic vector-based leader sequence (ΔG = −6.8 kcal/mol) lacking a TRβ1 UTR (see Materials and Methods). We found that the basic luciferase expression rates were the highest (24.09% of the control, p<0.001) when driven by the most weakly folded variant A, possessing the lowest (negative) value of Gibbs energy (ΔG = −69.0 kcal/mol, Fig 1b). In contrast, luciferase expression from plasmids containing variant G (ΔG = −127.0 kcal/mol) and the most folded variant F (ΔG = −128.9 kcal/mol) was strongly inhibited (3.00% and 4.03% of the control for variant G and F respectively, p<0.001). Similar effects were previously reported in human placental JEG-3 choriocarcinoma cells [22] and in Caki-2, a human clear cell renal cell carcinoma line [23]. To check whether the different luciferase protein levels resulted from changes in levels of particular transcripts, we quantified luciferase mRNAs after 6h of the coupled transcription-translation reaction. Real-time PCR revealed no significant differences in luciferase transcription rates driven from the tested variants A-G (Fig 1a). These results are consistent with previous observations in Caki-2 cells [23] and in another *in vitro* translation system based on rabbit reticulocyte lysates [22].

### Correlation between Gibbs energy and translation efficiency

Although multiple bioinformatic tools for the analysis of higher-order structures of RNA are available, their utility in predicting the effects of translation -silencing or -enhancing *cis*-acting elements on the levels of protein expression is limited [35]. These elements may require specific secondary and tertiary folding to exert their normal function and may regulate the translation of downstream sequences independently of their nucleotide composition and Gibbs energy (ΔG) status [4, 36]. Therefore, we investigated whether the Gibbs energy of TRβ1 5’UTRs (S1 Table) could correlate with 5’UTR-controlled translation efficiency of a downstream ORF. High Pearson’s correlation coefficient r^2^ = 0.9746 (p<0.005) showed that protein levels are strictly dependent on Gibbs energies of the TRβ1 5’UTRs (Fig 1). The correlation also resulted in an exponential equation (y = 140.46_*_e^0,0307*X^, Fig 1c) that could serve for theoretical prediction of the translation rate of any TRβ1 5’UTR variant. An example application of this equation is shown in S2 Table. Finally, the correlation allowed us to use ΔG-based algorithm derived from the dGenhancer calculator for an automatic design of oligonucleotide *trans*-acting factors (S1 Appendix).

### Prediction of *cis*-acting-elements of high regulatory importance

**Fig 2:**
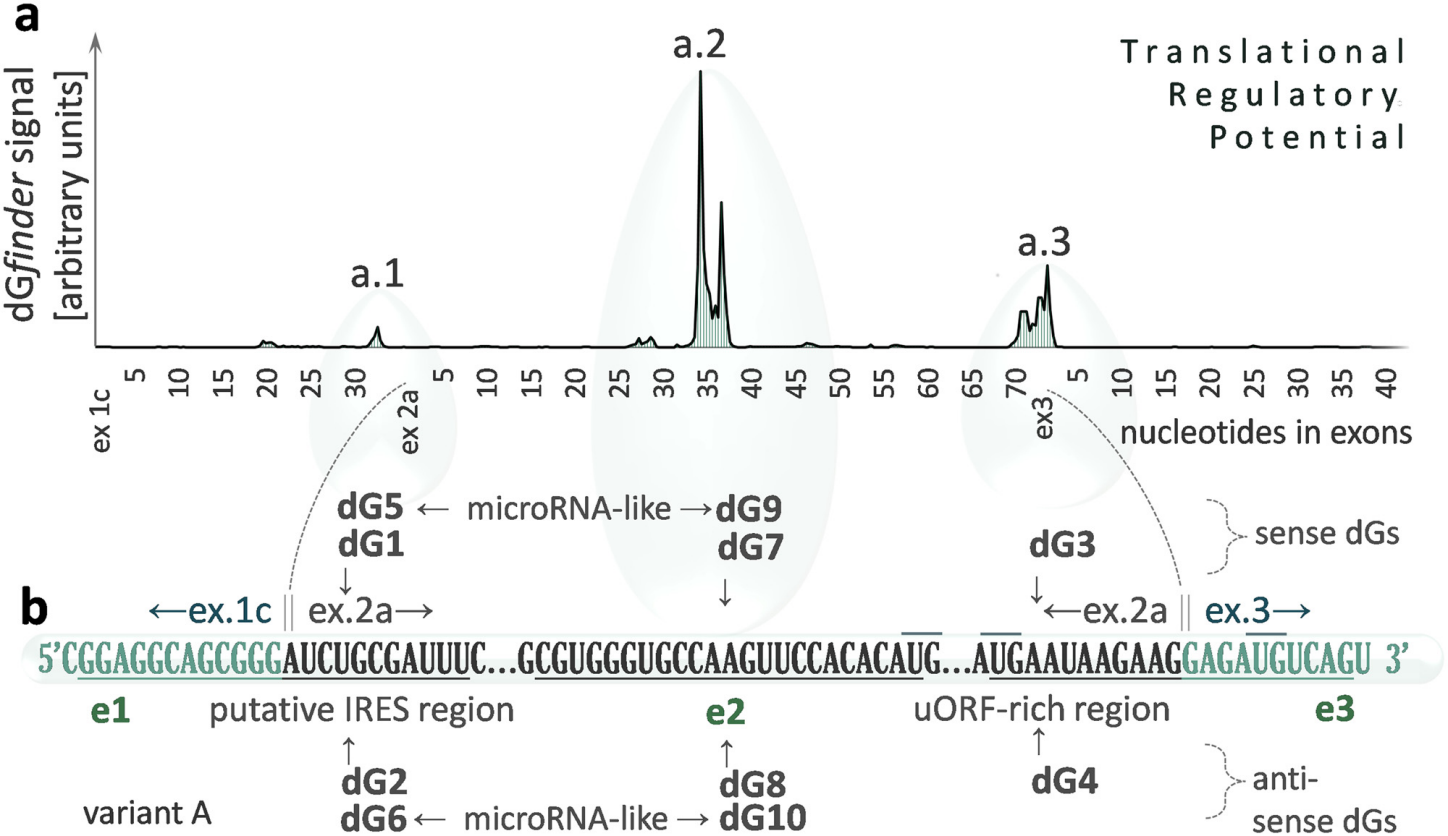
dGoligo recognition sites. (**a**) *Cis*-acting elements (e1, e2, e3) of variant A of TRβ1 5’UTR determined by dGenhancer, which indicates signal maxima (a.1, a.2 and a.3) corresponding to the 5’UTR fragments with the highest translational regulatory potential. The signal intensity represents transformed mean of 6 consecutive changes in Gibbs energy (ΔG) observed among 5’UTR sequences containing artificial SNPs. The SNPs were used as a theoretical model to calculate which sequence fragments (within the UTR) can change ΔGs (of the UTR) the most, thereby affecting the translational potential of the 5’UTR. (**b**) dGoligo (dGs 1–10) targets (e1, e2, e3) in TRβ1 5’UTR are shown as underlined sequences. dGs are presented above (sense) and below (antisense) the TRβ1 5’UTR A. Each dG shares homology with the TRβ1 5’UTR, targeting one of the indicated sequences: a putative IRES site on ex1c/ex2a junction (underlined) targeted by dG1, dG2, dG5, dG6, a sequence containing multiple alternate AUGs (uORFs-rich region) and located on ex2a/ex3 junction targeted by dG3, dG4 or a sequence in the middle of exon 2 targeted by dG7 dG8, dG9 and dG10. All dG*s* were designed as complementary pairs of antisense strand (dG2, dG4, dG6, dG8, dG10) directly recognizing the indicated region and sense strand (dG1, dG3, dG5, dG7, dG9) that can bind to distant sequences that interact through complimentary base-pairing with the indicated region (S1 Fig). Oligonucleotides dG5, dG6, dG9 and dG10 were synthesized as microRNA-like oligonucleotides with 3-nt insertion in the middle of their sequences.

Since most of the alternatively spliced variants of TRβ1 5’UTRs were shown to have strongly folded, translation-inhibiting sequences [22, 23], further study was performed to estimate their translational potential and find a method that could release the *potential* to enhance protein synthesis. We aimed to identify sequences within TRβ1 5’UTRs that could be of particular importance in this process. At first, structures of the TRβ1 5’UTR variants A and F (S1 Fig) were drawn with RNAstructure version 5.2 [37] to determine the most stable secondary structures accompanied by the most negative ΔG. These folding predictions allowed us to identify elements that are likely to be required for secondary and tertiary folding of the 5’UTRs. Then, the elements were compared with publicly known *cis*-acting sequences of IRESite database [38] that allowed us to identify common sequence motifs of possible functional importance. We selected a hairpin sequence within a previously reported domain containing a putative IRES, which has been identified before in the TRβ1–5'UTRs [23], and a sequence conserved among all TRβ1 5’UTR variants with multiple alternate AUGs [22] (Fig 2). To check functional properties of the putative IRES site, we performed a simple test in Caki-2 cells, cultured in serum-deprived medium, which has been reported to switch between cap-dependent and cap-independent (IRES-mediated) translation [5, 12]. We found that protein synthesis rates after serum starvation resulted in higher luciferase activity from plasmid containing the TRβ1 IRES site (pGL3-A) [22] compared to the control (pGL3-control) [22] without the IRES sequence (S2 Fig).

Finally, the manually selected translation -enhancing element e1 (IRES) and translation -inhibiting element e3 (uORFs, Fig 2) were compared with automatically determined elements identified by the dGenhancer. The calculator works on the basis of ΔG changes observed among *in silico* generated 5’UTR sequence variants that differ in a single nucleotide substitution (SNP) altering overall ΔG of the sequences. These artificial variants were created by substitution (base by base) in each nucleotide position of the 5’UTRs (S1 Appendix). Comparing these two approaches we found that the manually and automatically determined elements of the 5’UTRs are fairly similar with one exception of the strongest signal of dGenhancer showing an additional translation-inhibiting element marked as e2 (Fig 2), located in exon 2a, which is present in all TRβ1 5’UTR variants. Identification of these *cis*-acting elements allowed us to design and synthesize specific oligonucleotide-based *trans*-acting factors, termed dGoligos (dGs, Fig 2, S3 Table), designed to recognize and bind the predicted 5’UTR sequences.

### dGoligo design and synthesis

**Fig 3:**
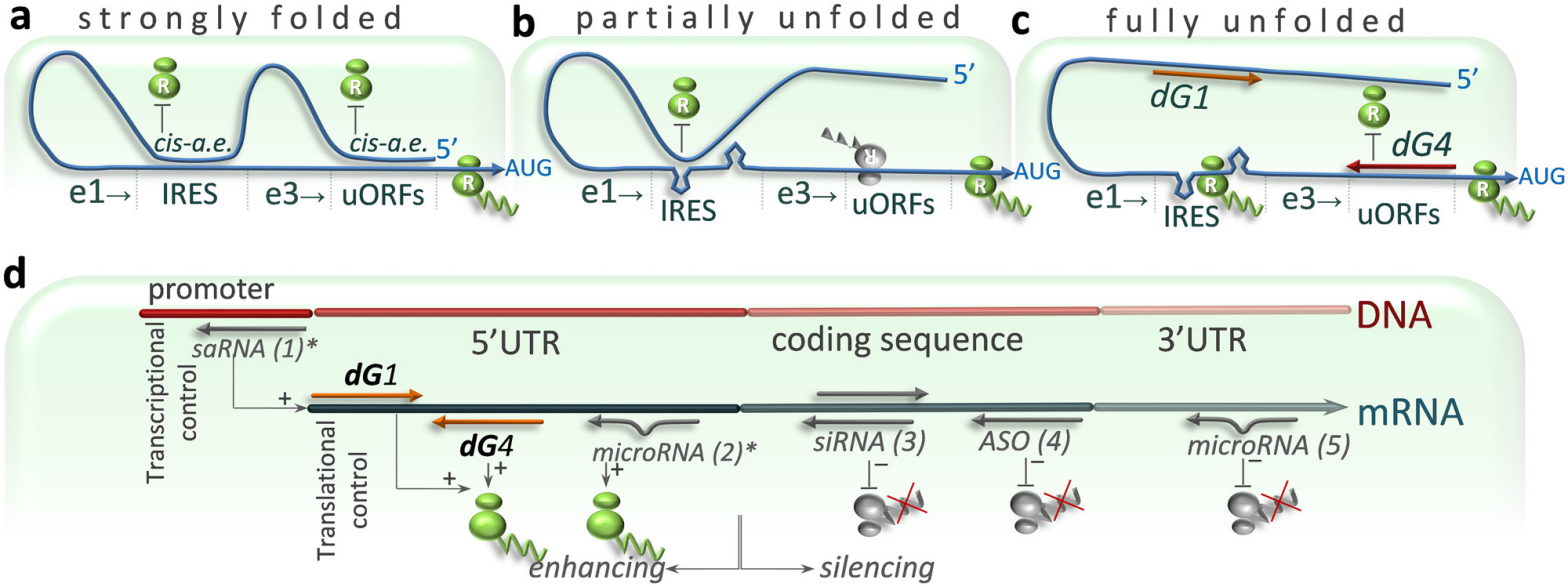
Folding states of TRβ1 5’UTRs. Translation efficiency of TRβ1 is dependent on folding states of its 5'UTR, which is proposed to be: strongly folded (**a**), partially unfolded (**b**) or fully unfolded following interaction with dG1 and dG4 (**c**). The 5’UTR is shown as curve ended by an arrow at AUG translation start codon. Two linked ovals assigned by letter R represent ribosome that may be blocked by distant *cis*-acting element (*cis*-a.e) or *trans*-acting factor (dG1 or dG4). AUG start codon is preceded by selected translation-regulating elements (e1 and e3). Translation-enhancing element e1 contains putative Internal Ribosome Entry Site (IRES) that may be involved in enhancement of cap-independent translation initiation. Translation-silencing element e3 contains upstream Open Reading Frames (uORFs)–rich region that can reduce translation initiation from correct AUG start codon due to simultaneous synthesis of truncated proteins originated from upstream AUGs (shown by inverted ribosome). **a**, Theoretical folding state of TRβ1 5'UTR characterized by the presence of highly-structured sequence that can block both: translation-enhancing e1 and translation-silencing e3, finally leading to only basal protein synthesis. (**b**) Another theoretical folding state described by partially unfolded 5’UTR with blocked e1 and unblocked e3, resulting in basal translation rates as well. (**c**) Proposed model of dG1 and dG4 -mediated enhancement of translation efficiency, wherein antisense dG4 could lead to repression of uORFs within e3, whereas binding of sense dG1 to a distant sequence (usually folded with e1) could release this translation-enhancing region, allowing for protein over-expression (additional description is given in S4 Fig). (**d**) dGoligo (dG) targets on mRNA. Locations of dG binding sites are shown in the context of typical targets of the most known small interfering RNAs. microRNA (2, 5), siRNA (3), saRNA (1) and ASO (4), are shown by short grey arrows. Newly described interactions that may result in up-regulation of gene expression are indicated by asterisk*.

We next evaluated whether we could selectively alter the Gibbs energy-dependent secondary structure formation of TR1 5’UTRs using oligonucleotide—based *trans*-acting factors. We synthesized a set of DNA oligonucleotides (dGoligos) directed to interfere with previously identified TRβ1 5’UTR *cis*-acting elements. High translational regulatory potential was defined as the potential of the translation-regulating elements to enhance protein synthesis from low to high rates. This regulation is thought to be controlled by distant *cis-* or *trans*-acting factors specifically binding to the regulatory elements (Fig 3d). A putative IRES [23] site and a sequence containing multiple alternate AUGs [22] were targets for dGoligos (dGs) dG1, dG2, dG5, dG6 and dG3, dG4 respectively (Fig 2). dG7 and dG8 were designed to target a sequence located in the middle of exon 2a, identified by the dGenhancer to have the highest regulatory potential. For *in vitro* assays, dG*s* were synthesized as sense-, antisense- or microRNA-like DNA oligonucleotides (S3 Table). 2’-O-methyl RNA-modified derivatives were synthesized for *in vivo* assays.

dG-mediated linearization of 5’UTRs was predicted to disturb inhibitory structures and/or liberate translation-enhancing elements. These proposed functions of the synthetic oligonucleotides were likely to be required for structural rearrangement of the 5’UTRs into their translationally active conformation (Fig 3) that facilitates interaction with naturally occurring elements directly controlling final protein levels.

### Translation-enhancing dGoligos targeting TRβ1 5'UTRs

The influence of dGoligos on translation efficiency was studied in coupled *in vitro* transcription-translation reactions using plasmids containing the least (A) and the most (F) folded variant of TRβ1 5’UTR cloned upstream of luciferase reporter. Effects of each dGoligo on protein synthesis were assessed in a translation-enhancing assay. Levels of luciferase mRNA and protein (luciferase activity) expressed from plasmid without or with dGoligo supplementation were measured by Real-Time PCR and luminometry. Maximum luciferase activity was observed after 6h (S3 Fig) and this time point was chosen for subsequent analyses. No statistically significant differences in luciferase mRNA levels were observed between control and plasmid constructs.

To eliminate the effects of possible non-specific dG-plasmid interactions, all transcription and translation measurements for both pKS-A and pKS-F plasmids were standardized to mRNA levels driven from control plasmid with a short irrelevant vector-based leader sequence that contained no specific dGoligo binding sites (pKS-control).

**Fig 4:**
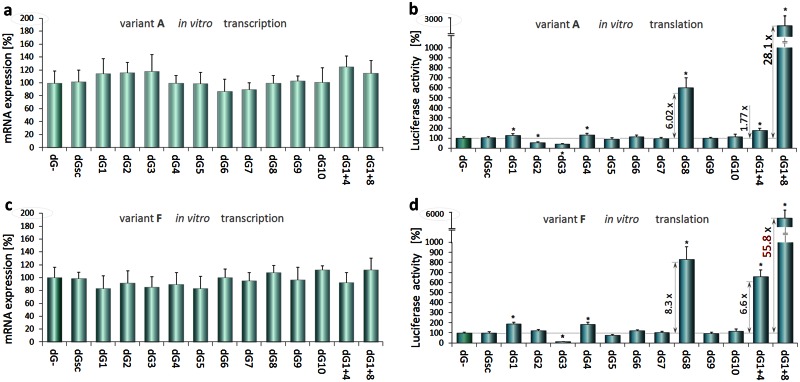
dGoligo-mediated gene expression changes under *in vitro* conditions. Effects of each DNA oligonucleotides dG1-dG10 (S3 Table), on *in vitro* transcription of luciferase reporter constructs (panels **a**, **c**) and translation efficiency (**b**, **d**), using pKS-A (a, b) or pKS-F plasmid (c, d). Data normalized to control (dG-) containing pKS-A or pKS-F without addition of dGoligo. Scrambled control (dGsc) had no effect on transcription or translation. The strongest enhancing effects on luciferase activity were obtained by combining dG1+dG8 (28.10-fold from pKS-A and 55.80-fold from pKS-F). Results from three independent experiments performed in triplicates are shown as mean % mRNA (a, c) or luciferase activity (b, d) ± SD. Data analyzed by ANOVA followed by Dunnett’s multiple comparison test. *p<0.001 vs. control.

**dG1 and dG2** were synthesized as a pair of complementary, sense- and antisense- DNA oligonucleotides. dG1 shares sequence with the most stable secondary structure of the translation-enhancing element e1 (Fig 2) containing a putative IRES site [23] (S4g1 Fig) while sequence of dG2 is complementary to this region. As a result, dG1 increased translation efficiency over 1.29-fold when using pKS-A and 2.90-fold in case of the use of pKS-F (p<0.001, Fig 4b and 4d), while dG2 decreased the protein levels by 1.80-fold for pKS-A and did not alter translation for pKS-F, probably due to the lack of 3’-end of exon 1c in the variant F (S1b Fig). Since the sense dG1 has the same sequence as e1 element of the 5’UTR, it can interact with and block the homologous distant mRNA sequences (*cis*-acting elements) (Fig 3a) that can fold with the e1 domain [23] and lead to its repression. Thus, dG1 was designed to release the domain allowing for appropriate folding of this sequence that appears to be required for efficient translation. Antisense dG2, complementary to e1 sequence, was designed to bind this region directly, preventing formation of an active sequence conformation (S4 Fig).

**dG3** (sense) and complementary **dG4** (antisense) were designed to target sequence at ex2a/ex3 junction of TRβ1 5’UTR (e3 in Fig 2) that contains numerous upstream translation start cordons—uAUGs (S4h1 Fig). The sense dG3 has the same sequence as the uORFs-rich domain of the 5’UTR, and thus may interact with distant *cis*-acting elements (Fig 3a), which normally can fold with uAUG-rich domains [22] and act as uORF inhibitors allowing for more efficient translation from the canonical start codon. Thus, dG3 was designed to release the uORFs-rich domain of the mRNA, and was expected to facilitate initiation of translation from upstream AUGs (S4h1 Fig) resulting in reduced initiation of protein synthesis from the correct AUG start codon (Fig 4b and 4d). By contrast, antisense dG4 was designed to bind the mRNA sequence containing the uAUGs to render the uAUG-rich region inaccessible for the translation machinery (S4b1 Fig), resulting in enhanced translation initiation from the correct AUG start codon. These predictions were supported by results showing that dG3 decreased translation efficiency by 2.40-fold for pKS-A and 7.25-fold for pKS-F (p<0.001), whereas addition of dG4 increased translation efficiency by 1.33-fold for pKS-A and 1.86-fold for pKS-F (p<0.001). The findings suggest that blockade of alternate uAUGs is important for efficient protein translation and are consistent with results showing that initiation codons located upstream of the correct start codon of the TRβ1 can markedly affect the efficiency of protein synthesis [4, 22]. The translation-enhancing action of dG4 could also be explained using a model of enhancement of ATF4 translation in stress conditions, which can *switch off* inhibitory uORFs by increasing the time of 5’UTR scanning [4]. This allows for ribosomes to *bypass* the uORFs and find the correct ATF4 start codon in the Kozak consensus sequence [4, 13]. Our results show that binding of dG4 to TRβ1 uORFs-rich region forms a double stranded sequence that possibly slows down the scanning machinery. Thus, the use of dG4 may delay translation initiation, as it is observed in stress conditions, leading to enhanced levels of correct proteins. Moreover, the uORF-regulated translation initiation in stress conditions is found to be accompanied by higher translation rates of IRES-containing mRNAs [4, 13]. Indeed, our *in vitro* experiments showed that combined addition of sense dG1 and antisense dG4 increased luciferase activity by 1.77-fold (p<0.001, Fig 4b) from pKS-A and 6.58-fold from pKS-F (p<0.001, Fig 4d). The translation-enhancing effect could result from simultaneous release of the translation-enhancing element (e1) [23] and block of the uORFs-rich region [22] (Fig 3c). These results may also suggest that strongly folded variant F could be characterized by a higher translational regulatory potential (S2 Table and S5 Fig).

Furthermore, analysis of **dG7** and **dG8**, designed on the basis of a *cis*-acting element detected by dGenhancer (S3 Table), revealed that **dG8** enhanced translation by 6.02-fold and 8.30-fold for pKS-A and pKS-F respectively, whereas sense **dG7** had no significant effect (Fig 4a and 4b). Interestingly, a combination of antisense dG8 and sense dG1 enhanced luciferase activity over 28.1- (pKS-A) and 55.8-fold (pKS-F) (p<0.001). These effects reinforce the finding that exon 2a is conserved in all TRβ1 alternatively spliced 5’UTR variants and suggest an important role in translation control from this locus. Thus, blocking of exon 2 with complementary antisense **dG8** resulted in the strongest enhancement of translation, indicating that the *cis*-acting element at this site (e2) is not affected by other distant sequences of the 5’UTRs and has a key inhibitory role in translational control of TRβ1. These findings support the hypothesis that dGenhancer may be used to identify ΔG-dependent, translation-regulating elements in 5’UTRs that could be targeted by dGs to alter their Gibbs folding energy and regulate the translation efficiency. Finally, the data suggest a role for the multiple alternatively spliced TRβ1 5’UTRs. Strongly folded variants (including variant F) may serve as a reservoir of less-active mRNAs that could be recruited to increase translation efficiency at times of cellular stress, for example, by the use of specific *trans*-acting factors such as ncRNAs. Interestingly, bioinformatic analysis of microRNA target sites within TRβ1 untranslated regions revealed that hsa-miR-211 could potentially target both TRβ1 3’UTR and 5’UTR (S6a and S6b Fig) and binding of this non-selective ncRNA could at least affect secondary structures of the UTRs. Indeed, 2’-O-methyl RNA modified hsa-miR-211 enhanced TRβ1 5’UTR-mediated translation by 1.95-fold in Caki-2 cells (S6c Fig).

### dGoligo controls and binding capacity

All the results presented above show that, in contrast to translation-enhancing dGs, their complementary control partners (antisense dG2, sense dG3 and sense dG7) had no or opposite effects (Fig 4b and 4d), thus confirmed target site-specific action of sense dG1, antisense dG2 and antisense dG8. The fact that both sense and antisense oligonucleotides directed to bind 5’UTRs seriously altered translation levels gives a new insight into the nature of these molecules and indicates that this action may depend on specific properties of a target *cis*-acting element. Interestingly, these results also suggest that sense oligonucleotides, used in numerous studies as a control to antisense nucleotides (ASOs), could actually interact with distant *cis*-acting elements, significantly changing translation efficiency as it was shown in our study (Fig 4d).

To check whether the sequence structure of the dG*s* has an impact on their function we synthesized **mismatched control dG5** and **control dG6** sharing the same sequence with dG1 and dG2, respectively, but containing a 2–3 nucleotide long insertion in the middle of both oligonucleotides (Fig 2 and S3 Table). Upon binding target sequence, these additional nucleotides should form a loop that mimics metazoan microRNA structure and prevents perfect base pairing with target TRβ1 5’UTR. By mutating the dGs instead of their target sequences, we avoided problems with undesirable loss of functional properties of investigated 5’UTR *cis*-acting elements [3, 17]. Since numerous studies suggest that translationally active conformation of the UTR variants is of greatest importance for the UTR-mediated translational control, it seems our strategy was the best choice for subsequent control reactions. In addition, *in vitro* transcription-translation assays were performed in wheat germ extract and, as reported, plant microRNAs require nearly perfect base pairing with the target RNA to exert RNAi related effects [39]. Therefore, mismatched dG5 and dG6 served as mutated controls for other dG*s* (S4e1 and S4f1 Fig) and were expected not to exert any possible RNAi effects in the wheat germ translation system. As a result, neither the control sense dG5 nor control antisense dG6 altered translation levels (Fig 4b and 4d) that may provide a proof for selectivity of other fully complementary dG*s*. Similar microRNA-like controls were designed on the basis of another pair of dG*s* (dG7, dG8) and termed **dG9** and **dG10** (S3 Table). The use of these oligonucleotides revealed no effects on translation, supporting the observation that in the used plant-derived translation system, antisense-like dG*s* need full complementarity to affect gene expression [39].

Additional **scrambled control** (dGsc) with a random sequence (S3 Table) was also shown to have no effect on luciferase activity (Fig 4). dG binding assays revealed high binding capacity of all tested antisense-like dGs that were complementary to pKS-A transcripts (S7 and S8 Figs). Although sense-like dGs shared the same sequence with the variant A of the TRβ1 5’UTR (pKS-A), they were able to bind RNA as well, however, with a lower capacity when compared to the antisense dGs. At the same time, the binding of the scrambled control was undetectable (S7 Fig). These results may confirm our assumption that sense dGs can bind, at least partially, to the distant inhibitory sequences of the TRβ1 5'UTR, releasing translation-enhancing elements normally blocked by secondary structures in a translationally less active transcripts (Fig 3c).

### Protein up-regulation induced by p16 5'UTR-specific dGoligos

**Fig 5:**
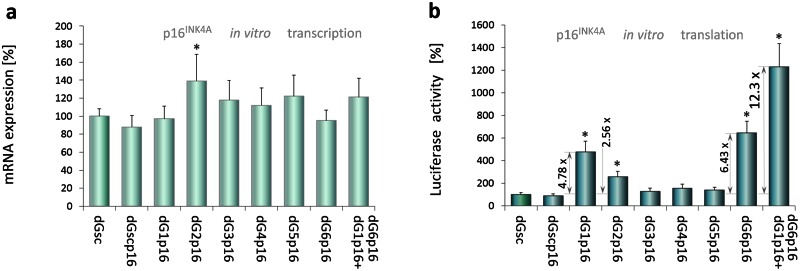
dGoligo-mediated upregulation of *CDKN2A* expression. PCR-amplified linear luciferase expression construct containing 5’UTR of p16^INK4a^ (*CDKN2A*) was generated (S1 Appendix) and used as a template in coupled transcription-translation assay that was performed as described in experiments with TRβ1 5'UTRs. (**a**) Luciferase mRNA levels after 6-hour *in vitro* reaction of the linear construct with a DNA-based dGoligos dG1p16—dG6p16, dG1p16+dG6p16 or dGscp16 (S3 Table) targeting the p16^INK4a^ 5'UTR. (**b**) Luciferase activity as a measure of dG-mediated translational control. All data are shown as mean % mRNA (a) or luciferase activity (b) ± SD. Data were analyzed by ANOVA followed by Dunnett’s multiple comparison test. *p< 0.001 vs. control.

To test whether our approach could be applied to enhance expression of another gene, we used published sequence data [21] as well as dGenhancer calculator to design dG*s*, specifically targeting p16^INK4a^ 5'UTR (NCBI Ac.: NM_000077.4), a transcript of *CDKN2A* tumor suppressor. In this *in vitro* study, dG-mediated regulation of protein synthesis was tested using PCR-amplified linear expression construct containing T7 promoter, p16^INK4a^ 5’UTR and the coding sequence of luciferase allowing for fast and reliable measurements of protein levels (S1 Appendix). The effects of each DNA-based dG*s* dG1p16-dG6p16 (S3 Table) were measured using coupled *in vitro* transcription-translation assay (Fig 5). Results from semi-quantitative real-time PCR, performed with luciferase specific primers (S4 Table), and measurements of luciferase activity revealed that negative control (dG-), scrambled control (dGscp16), dG5p16 and microRNA-like dG3p16 and dG4p16 had no effects neither on transcription nor translation efficiency. These results are in agreement with our data, including those showing no effects of microRNA-like DNA dGs in wheat germ lysates (Fig 4). Sense dG1p16 and antisense dG2p16 were designed on the basis of an element e1 (S1c Fig) containing an IRES sequence [21]. In samples supplemented with sense dG1p16 we observed unchanged transcription that was accompanied by strong translation-enhancing effect (4.78-fold, p<0.001). Similarly, dG2p16 elevated protein levels by 2.56-fold (p<0.001), however, this particular result could be a consequence of higher mRNA levels (1.3-fold, p<0.001). These results may indicate that apart from the explicit dG-mediated translation-enhancing effects, confirming findings obtained with TRβ1 5'UTRs, some dG*s* can influence transcription machinery as well, thereby resembling the action of saRNAs [29, 30]. Using a combination of dG1p16 and dG6p16 (Fig 5) we observed over 12.30-fold increase in luciferase activity that is in accordance with previously observed effects triggered by a mixture of sense and antisense dGs: dG1+dG8 or dG1+dG4 targeting TRβ1 5'UTRs. All the results were normalized to control (dG-). Data from three independent experiments were performed in triplicate and analyzed by ANOVA followed by Dunnett’s multiple comparison test. *p< 0.001 vs. control.

### dGoligo-mediated translation-enhancing effects in Caki-2 cells

**Fig 6:**
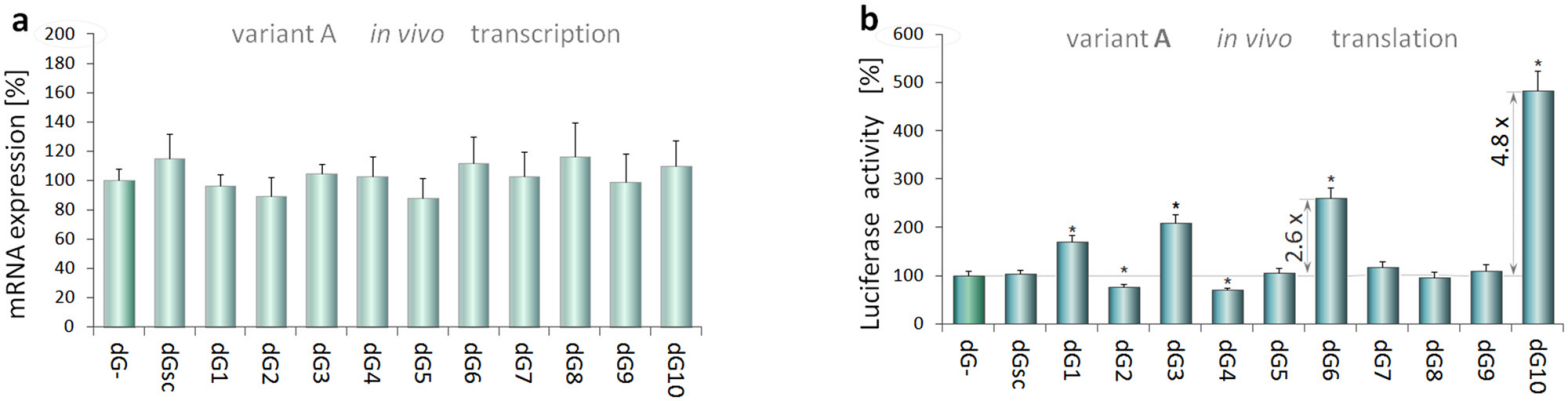
dGoligo-mediated gene expression changes in Caki-2 cells. Effects of 2’-O-methyl RNA oligonucleotides on luciferase transcription (panel **a**) and translation (**b**) in Caki-2 cells transfected with pGL3-A. MicroRNA-like dG10 and microRNA-like dG6 exerted the strongest translation-enhancing effects in Caki-2 cells (4.83-fold and 2.60-fold respectively). Results from three independent experiments performed in triplicates are shown as mean % mRNA (a) or luciferase activity(b) ± SD. Data analyzed by ANOVA followed by Dunnett’s multiple comparison test. *p<0.001 vs. control.

To test our *in vitro* data in a cellular context, similar experiments were performed in Caki-2 cells using TRβ1 5’UTR A (pGL3-A) and appropriate dG*s* (Fig 6). Although unmodified deoxyoligonucleotides can display some *in vivo* activity, they are subject to rapid degradation by nucleases and are of limited utility in mammalian cells [40]. Therefore, we synthesized nuclease-resistant, 2'-O-methyl RNA modified oligonucleotides, which do not activate the RNase H pathway [41]. Fig 6b shows that each dG differently regulated reporter protein synthesis. After transfection the cells with the DNA-based dG*s* targeting variant A of TRβ1 5’UTR there was no significant effect on translation of luciferase reporter protein (S9 Fig). By contrast, RNA-based dG*s* showed increased translation efficiency between 1.7–2.1-fold (dG1, dG3), while the action of dG2 and dG4 resulted in 1.3–1.4-fold decrease in the reporter protein levels (Fig 6b). Surprisingly, antisense microRNA-like dG6, which was previously used as a mismatched control in *in vitro* assay, resulted in 2.6-fold increase in luciferase activity, whereas sense dG5 had no significant effect on translation when compared to control without any dG (p<0.01). The similar effects were observed when using microRNA-like dG9 and dG10 (1.09- and 4.8-fold respectively). These results showed a difference between the *in vitro* and *in vivo* studies, wherein the TRβ1 5’UTR targeting microRNA-like dG*s* exerted the strongest enhancing effects on translation in Caki-2 cells. The observed difference compelled us to introduce an additional 2'-O-methyl RNA modified scrambled control (dGsc) with an irrelevant (random) sequence that was shown to produce no change in luciferase activity, thus, confirming the specificity of the *in vivo* dG action. All the results of the luciferase activity after dG supplementation were normalized to control pGL3-A plasmid (mock transfected group). Data from three independent experiments were performed in triplicate. The Shapiro—Wilk test was used to determine normality of data distribution. Normally distributed data were analyzed using ANOVA followed by Dunnett’s multiple comparison test.

### Over-expression of endogenous TRβ1 proteins in dGoligo-treated cells

**Fig 7:**
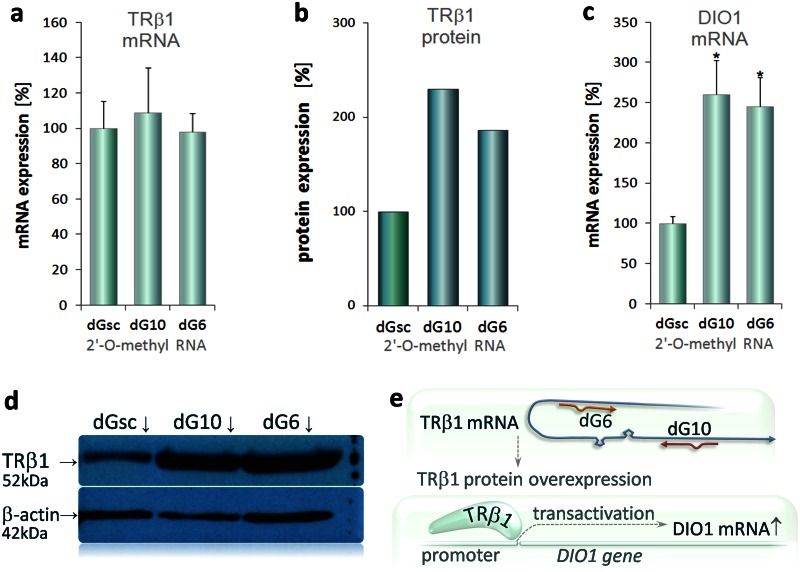
Effects of selected dGs on expression of endogenous TRβ1 in Caki-2 cells. Caki-2 cells were transfected with 2’-O-methyl-modified RNA-based dG6, dG10 or scrambled control—dGsc and cultured (without any plasmid) according to the procedure used in translation-enhancing assay (Materials and Methods). dGs were selected on the basis of previously obtained results (Fig 6). Expression of TRβ1 mRNA (**a**), protein (**b**) and DIO1 mRNA (**c**) are shown in upper panel. Semi-quantitative real-time PCR was performed for TRβ1 (exon 2–3) and DIO1, as described before [23]. Data from three independent experiments were performed in triplicate and shown as mean values ± SD. Statistics were calculated using t-test to compare cells transfected with dGs vs. dGsc. *p<0.001. (**d**) An example western blot for TRβ1 and β-actin is shown in lower panel. Each band (dGsc, dG10, dG6) represents sample combined from nine protein lysates. Relative density of bands was quantified by densitometry and TRβ1 protein levels were normalized to β-actin. (**e)** A simplified model of dG-mediated upregulation of endogenous TRβ1 protein, which has been demonstrated before to act as a transcription factor activating transcription of multiple genes including type 1 iodothyronine deiodinase (DIO1).

Translation-enhancing properties of selected dGs were confirmed in Caki-2 cells, where transfection with dGs resulted in increased levels of endogenous TRβ1 protein and its downstream target—type 1 iodothyronine deiodinase DIO1 [8, 23] (Fig 7). In this part of the study, Caki-2 cells were transfected with 2’-O-methyl-modified RNA-based dG6, dG10 or scrambled control—dGsc and cultured (without any plasmid) according to the procedure used in transcription and translation assay. dGs were selected on the basis of previously obtained results (Fig 6). Semi-quantitative real-time PCR for TRβ1 (exon 2–3) and DIO1, as well as western blot for TRβ1 and β-actin (Abcam plc, Cambridge, UK) were performed as described before [23]. Relative density of bands was quantified by densitometry and TRβ1 protein levels were normalized to β-actin. Fig 7b and 7d show that the most efficient enhancement of translation was achieved by action of antisense, microRNA-like dG10, which upregulated the endogenous TRβ1 protein synthesis by over 2.3-fold, whereas TRβ1 mRNA levels remained unchanged (p<0.001, Fig 7a). These results may provide evidence that translation of endogenous TRβ1 can be enhanced by dGs resulting in modification of the functional response, as evidenced by over 2.5-fold increase in expression of the DIO1 target gene (p<0.001, Fig 7c). Data from three independent experiments were performed in triplicate and shown as mean values ± SD. Statistics were calculated using t-test to compare cells transfected with dGs vs. dGsc. *p<0.001.

## Discussion

These studies demonstrate that specific enhancement of gene expression can be achieved at the level of protein translation. We found this phenomenon to be triggered in a specific manner by an exogenous synthetic small enhancing oligonucleotide—dGoligo (dG) targeting a specific 5'UTR *cis*-acting element.

### Targeting 5’UTRs could be a novel way to control protein levels

As previously demonstrated, alternative splicing of TRβ1 5’UTR variants is impaired in human clear cell renal cell carcinoma (ccRCC) and differential expression of multiple mRNA variants is accompanied by varying levels of TRβ1 protein expression [23]. Although the functional significance of these observations is not known in ccRCC, aberrant expression of alternative 5’UTRs has been shown to contribute to carcinogenesis mediated by silenced tumor suppressors [42] or activated oncogenes [43]. In the light of complex secondary structures of low copy number TRβ1 5’UTRs including variant F [23] and evidence for selective protein synthesis of some alternatively spliced mRNA variants in oxygen deprived tumors or metastatic cancers [13, 16], it has been suggested that the sequence diversification of TRβ1 5’UTRs could play an important role in controlling *THRB* gene expression and this may influence tumor progression [23, 44]. Thus, the reported lack of correlation between the mRNA and TRβ1 protein levels in ccRCC [23] raised the hypothesis that the observed impairment may result, at least in part, from differing translational efficiencies of the TRβ1 5’UTR variants. This hypothesis is supported by the correlation observed between the *in vitro* translation efficiency of each 5’UTR and its Gibbs energy (Fig 1c), resulting in the aim to evaluate whether translation efficiency could be altered specifically by affecting folding Gibbs energy (ΔG). To investigate further, we used oligonucleotide—based *trans*–acting factors termed dGoligos (S3 Table) to selectively target TRβ1 5’UTRs and change the Gibbs energy-dependent secondary structure formation of the 5’UTRs (Fig 2). Since a misfolded conformation of mRNA *cis*-acting domains could result in either enhanced or reduced protein translation [4, 5], direct binding to these domains (in case of antisense-like dG*s*) or binding to distant *cis*-acting sequences folding with these domains (sense-like dGs) may enhance or repress protein synthesis (Fig 3). To find ΔG-dependent, translation-regulating domains we used a bespoke dGenhancer calculator, which allowed us to design the most effective, translation-enhancing dGs (S1 Appendix). However, this version of the calculator is unable to show ΔG-independent, functionally-active elements including IRESs, therefore it should be used together with other available databases of *cis*-acting elements.

### Strongly folded 5'UTRs have higher regulatory potential

In this study, the strong enhancement of translation was achieved by coupled action of sense dG1, designed to unblock a translation-enhancing element (e1), and antisense dG4, directly binding to an inhibitory region (e3, Fig 2). When both dG*s* were added, 1.77-fold and 6.58-fold increases in translation efficiency from weakly folded 5’UTR variant A and strongly folded variant F respectively were observed (Fig 4b and 4d). At the same time, the basal translation level (control without any dG) (Fig 1b) of variant A (24.09% of control) was 5.96-fold higher when compared to variant F (4.03% of control), suggesting that the folded variant F could possess higher translational regulatory potential that was triggered by dG1 and dG4 (S5 Fig). These results suggested the hypothesis that mRNAs containing strongly folded 5’UTRs may constitute a pool of translationally non-active or less-active transcripts that could be recruited to translation through interaction with naturally occurring small RNAs [18, 34], which may interfere with mRNA 5’UTRs in the same way as our dG*s*. This hypothesis is supported by the previously reported observation that cellular microRNA miR-10a can interact with the 5’UTR of mRNAs encoding ribosomal proteins that results in enhancement of their translation and may be implicated in tumor invasion and metastasis [45]. Here, we showed translation-enhancing effects of synthetic, TR1 5'UTR-specific, microRNA-like dGs (Fig 6), however, we also found naturally occurring microRNA hsa-miR-211 to have target sites in both TRβ1 3’UTR and 5’UTR (exon 2/3, S6 Fig). Furthermore, recent studies on 3’UTR-mediated gene silencing showed a correlation between microRNAs targeting 3’UTRs and 5’UTR structures, which can recruit RNA helicase eIF4A2, a key factor of eIF4F through which microRNAs function [18]. The authors have demonstrated that the eIF4A2 activity in 5’UTRs are critical for microRNA-mediated gene regulation as well as that mRNAs with weakly folded 5’UTRs are refractory to microRNA repression [18]. This report and our current results show that, in spite of low basal translation efficiency of mRNAs containing highly-structured 5’UTRs (Fig 1b), these regions alone or together with 3’UTRs have higher translational regulatory potential compared to unfolded 5’UTR variants (Fig 4b and 4d). It seems, therefore, that UTR-controlled translation-enhancing or -silencing phenomena could be triggered in response to exposure to available *trans*-acting factors that may lead not only to gene repression [9, 27] but also gene activation [30, 31].

### dGoligos can lead to over-expression of selected proteins

*In vitro* results revealed that both, sense and antisense dG*s* can trigger translation-enhancing effects that appear to be mostly dependent on a specific function of a 5'UTR *cis*-acting element. The action of sense dG1 was thought to increase protein synthesis by releasing translation-enhancing element e1 (Fig 3c) containing a putative IRES domain that has been identified before in the TRβ1–5'UTRs [23] and tested in Caki-2 cells (S2 Fig). The translation-enhancing action of antisense dG4 and dG8, which are complementary to a highly structured region containing multiple uAUGs, could be explained by linearization of their target sites and blocking the uAUGs-rich region to prevent from translation of alternative polypeptides (Fig 3c). This explanation is in agreement with a well-studied model of the selective enhancement of ATF4 protein synthesis during integrated stress response (ISR) [4, 13]. ISR can delay cap-dependent translation that makes uAUGs less attractive as start codons and allows ribosomes to scan through the inhibitory uAUGs to find the correct codon of ATF4 [4]. Similarly, antisense dG4 or dG8 could serve as a *trans*-acting factor making the TRβ1 uAUG-rich domain inaccessible for the translation machinery, thus, facilitating the ribosomes to start the synthesis at the correct AUG. These antisense-mediated effects might be supported by cap-independent translation, initiated at the IRES domain [12] that is released by sense dG1. Indeed, the most efficient translation was observed in the presence of sense dG1 and antisense dG8 that enhanced *in vitro* luciferase activity over 28.1 and 55.8-fold for variant A and F, respectively (Fig 4b and 4d). At the same time, transcription levels were noted to be unchanged (Fig 4a and 4b), suggesting that this regulation may differ from recently described RNAa phenomenon resulting in up-regulation of gene transcription, induced via promoter-specific activation [29, 30] or by promoter-directed antigene RNAs [31, 46].

In contrast to translation-enhancing dGs, their complementary partners (dG2, dG3 and dG7) had no or opposite effects (Fig 4). Moreover, neither mismatched control dG5 nor control dG6 altered protein levels in significant way (Fig 4b and 4d). Scrambled control with a random sequence was shown to have no effect on luciferase activity as well (Fig 4b and 4d), thus, confirming target site-specific action of dG1, dG4 and dG8.

In studying the translational regulatory potential of TRβ1 5'UTR variants we raised the question whether the observed translation-enhancing effects triggered by dGs are universal or TRβ1-specific. To check this out we designed dGs against the IRES identified within the p16^INK4a^ 5'UTR (*CDKN2A*) [21] and used the dG*enhancer* calculator to design dGs specifically targeting the ΔG-dependent, translation-regulating elements within this 5'UTR (S1 Appendix). The *CDKN2A* gene is frequently mutated or deleted in a wide range of tumors and produces at least three alternatively spliced variants encoding four distinct proteins [21]. An analysis of translation under the control of the p16^INK4a^ 5'UTR, which was incorporated into a PCR-amplified linear luciferase expression construct (S1 Appendix) revealed a 4.78-fold increase in protein levels and unchanged transcription rates after addition of dG1p16 (Fig 5). As was found for the dG1 unblocking IRES oligo in TRβ1 5’UTR (Fig 3), the sense dG1p16 can enhance translation via binding to distant sequences that may interact through complimentary base-pairing with the IRES region of the p16^INK4a^ 5'UTR. The strongest enhancing effects on luciferase activity (12.30-fold) were obtained by combining dG1p16 and dG6p16 (Fig 5) that is in accordance with previously observed in TRβ1 5'UTRs reactions translation-enhancing effects triggered by a mixture of sense dG1+dG8 or dG1+dG4 unblocking an IRES region (e1) and blocking translation-inhibitory element (e2 or e3). Although different constructs were used in this study, these results clearly confirm findings obtained *in vitro* with TRβ1 5'UTRs and show that dGs could be used as an universal tool controlling levels of selected proteins.

### microRNA-like dGoligos are more effective to enhance *in vivo* translation

These experiments were designed to investigate whether dG*s* can regulate protein translation in a cellular context. We used Caki-2 cells transfected with pGL3-derived plasmid [23] carrying TRβ1 5’UTR and downstream luciferase that allows for fast and reliable assessment of quickly changing translation rates in these cells after treatment with dGs. In contrast to results obtained *in vitro* with RNase H deficient wheat germ extracts, transfection of Caki-2 with DNA based dG*s* did not alter luciferase activity (S9 Fig), likely because unmodified deoxy-oligonucleotides are rapidly degraded by cellular nucleases [41], which can also switch off the translation in a non-specific way [15]. The use of 2'-O-methyl RNA -modified dG*s*, however, influenced the translation efficiency in these cells (Fig 6). Surprisingly, 2'-O-methyl modified, antisense, microRNA-like dGs: dG6 and dG10, containing a 3 nucleotide long insertion (loop) in the middle of their sequences (S3 Table), resulted in a greater than 2.6-fold and 4.8-fold increase in luciferase activity, respectively, whereas microRNA-like sense dG5 (complementary to dG6), sense dG9 (complementary to dG10) and scrambled control dGsc had no significant effect on the translation (Fig 6). All tested dGs did not affect mRNA levels suggesting that they could be involved specifically in translational control. These results are consistent with reports showing that some naturally occurring microRNAs can bind to 5'UTRs and regulate translation initiation [26, 45], however, their selectivity toward a single mRNA is thought to be low [27]. In contrast, synthetic micro-RNA like dGs with almost full complementarity to a target sequence and reduced positions of potential G:U wobble base-pairing were shown to have high binding capacity and selectivity toward the complementary sequence (S7c Fig).

### dGoligo-treated cells can enhance translation of a native protein

Translation-enhancing properties of selected 2’-O-methyl-modified dGs were confirmed in Caki-2 cells on translation of endogenous TRβ1 protein that has been reported to be a transcription factor controlling transcription rates of type 1 iodothyronine deiodinase DIO1 [8]. DIO1 transcript, therefore, served as an estimate for TRβ1 transcription factor activity, which was expected to be dependent on the TRβ1 protein levels [8, 23]. Our experiments showed that the cells (without any plasmid) transfected with microRNA-like dG10 over-expressed the DIO1 mRNA by 2.5-fold that was accompanied by 2.3-fold enhancement in translation of the endogenous TRβ1 protein (Fig 7). It has also been shown that the levels of this protein can be elevated even more using alternative methods of the dGs delivery [47]. All tested dGs had no impact on TRβ1 mRNA levels, and treatment with scrambled control (dGsc) unchanged transcription and translation rates. Therefore, the elevated levels of DIO1 mRNA may indicate higher transcription factor activity of TRβ1 [8, 23] in the dG-treated cells (Fig 7e) and may provide evidence that dGs can affect the functional response of the living cells.

### dGoligo may interfere with machinery of translational control

Although the exact action of dGs remains unknown, it is clear that binding of these oligonucleotides can affect secondary and tertiary structures of a target sequence that may result in altering its translation regulating properties (Fig 3). This action is considered to trigger subsequent mechanisms leading to translation-enhancing or -silencing effects [18, 29].

Antisense DNA oligonucleotides (ASOs) are widely used to suppress gene expression by inducing RNase H-mediated mRNA degradation of the target mRNA [48]. The DNA/RNA heteroduplexes are subsequently targeted for endonucleolytic cleavage by the RNase H, however, previous observations suggest that ASOs, which are usually used to target a coding sequence, may result in RNase H-dependent generation of stable mRNA cleavage fragments without 5’-cap, followed by expression of truncated proteins. The lack of the 5’-cap structure could further be bypassed by the cap-independent but 5’ end-dependent translation, initiated from an AUG start codon located a few nucleotides downstream of the 5’ end of the RNA fragment [48]. This mechanism of translation was observed *in vitro* and *in vivo*, albeit with severely reduced efficiency [48]. Translation of the cleavage fragments may also occur via direct binding of ribosomes to internal RNA secondary structures (IRESs) present on various cellular mRNAs, however, the IRES-mediated translation efficiency is condition-dependent [5, 13] (S2 Fig). These findings provide a rationale for understanding the translation of mRNA fragments generated by RNase H and could be considered *in vivo* as a potential mechanism of action of small enhancing oligonucleotides. They, as other ASOs, may interfere with the RNAse H pathway and subsequently generate RNA cleavage fragments [48] including transcripts with shorter, less folded 5’UTRs. However, it was also elucidated, that 2’-O-methyl sugar modifications result in an increased resistance to nuclease degradation [41, 49]. In addition, RNase H activity in wheat germ lysates has been reported to be markedly reduced in comparison to other mammalian-based translation systems [49]. Moreover, in our *in vitro* coupled transcription/translation experiments with dG*s*, the levels of transcripts after 6-hour reactions were unchanged (S3 Fig), suggesting that, indeed, RNAse H could not induce cleavage of dGoligo target sites and probably do not have strong impact on the observed over 58-fold (dG1 and dG8) enhancement of translation efficiency in the used *in vitro* system.

Comparing results from two different transcription-translation assays performed in the plant cell-free lysates and human cells (Figs 4 and 6), we considered whether dGs could be involved in RNAi/RNAa related phenomena. Unlike mammalian microRNAs, plant microRNAs require nearly perfect base pairing to induce the RNAi machinery [39]. Our results showed that neither microRNA-like dG5 nor dG6 altered *in vitro* protein levels in significant way (Fig 4b and 4d), indicating that when the assay is performed in the plant extract, a microRNA-like sequence loop introduced in the synthetic dGs can block their action. On the contrary to fully complementary sense/antisense-like dGs that we found to be the most effective in the plant system (Fig 4), the antisense microRNA-like dGs exerted the strongest translation-enhancing effects in Caki-2 cells (Fig 6). These findings are in agreement with distinct mechanisms of RNA interference in mammals and plants and could serve as an argument for involvement of dG-5'UTR dimmers in some elements of this machinery. Although our assumption needs to be studied in details, it can be supported by the known action of non-selective translation-enhancing microRNAs including miR-122 [26] or miR-10a [45] and a link between microRNA targets in 3’UTRs and 5’UTR structures that are thought to play an essential role in RNAi [18]. Recently discovered small activating RNAs (saRNAs) [28] can also trigger mechanisms leading to similar gene-enhancing effects, however, unlike our single stranded translation-enhancing dG*s*, saRNAs have been shown to be effective as double stranded transcription-activating molecules targeting promoter regions [29].

## Conclusion

In summary, this work presents the first evidence for gene-specific translation-enhancing effects triggered by small selective oligonucleotides termed dGoligos (dGs). These synthetic *trans*-acting factors were originally designed to alter Gibbs energy-dependent secondary structure formation of TRβ1 5’UTRs encoded by *THRB* suppressor gene. The applied approach allowed us for over 55.8-fold translational enhancement of reporter protein when dG1 and dG8 were used in coupled *in vitro* translation-transcription assay. Complementary *in vivo* study showed that dGs can enhance TRβ1–5’UTR -mediated translation up to 4.8-fold. Interestingly, this assay showed that protein can be more effectively synthesized when microRNA-like, 2'-O-methyl RNA antisense dG*s* were used. Furthermore, dGenhancer calculator, which allowed us to determine targets within TRβ1 5’UTRs, was also successfully used to design dGs enhancing translation of another *CDKN2A* tumor suppressor transcript, thus confirming the universality and potential of dGs to over-express selected proteins. The concept of this approach was based on our discovery that the most folded 5'UTR variants have higher translational regulatory potential that can be released to enhance translation efficiency by the use of specific dGs. They served as a molecular switch to translationally active conformation of the folded 5'UTRs. Taking together, this report would be the first showing a method for specific activation of translation-enhancing elements of high regulatory potential. This strategy may complement other available methods for gene expression regulation including gene silencing and may find its use in enhancement of genes frequently silenced in cancers or even in biotechnology of recombinant proteins.

## Materials and Methods

### Luciferase reporter constructs

*In vitro* studies were performed with pBluescript-KS(+)-derived plasmid vectors containing different TRβ1 5’UTR variants (pKS-A,-B,-C,-D,-E,-F,-G) or irrelevant leader sequence lacking any TRβ1 UTR (pKS-control) [22]. 5'UTRs were subcloned upstream of the luciferase reporter gene [22]. For *in vivo* analyses, we used pGL3-derived plasmid, carrying variant A of TRβ1 5’UTR (pGL3-A) [22], which was found to be the most predominant in kidney cells [23]. pGL3-control (without TRβ1 5’UTR) served as a control plasmid [22].

### Prediction of translation-enhancing elements

Two methods were used. *Manual* method allowed us to identify higher-order structures within 5’UTR *cis*-acting sequences (IRESs or uORFs stretches). Folding predictions from RNAstructure version 5.2, together with sequence analysis using NCBI tools were combined to select putative *cis*-acting elements containing the most stable secondary structures (the most negative ΔG). As a second method, dGenhancer—an excel-based calculator was used to automatically identify putative ΔG-dependent translation-regulating elements within 5'UTR sequences (S1 Appendix). The algorithms of the calculator were constructed to visualize ΔG changes after *in silico* introduced single nucleotide substitutions (SNPs) of the 5’UTR sequences. These artificial SNPs differently affected overall sequence ΔGs (Gibbs energies) that were drawn by the dGenhancer to show regions where substitution can alter ΔGs the most, indicating putative *cis*-acting elements with the highest translational regulatory potential. The software that implements the calculations can be accessed here: http://www.serwer1448847.home.pl/biotechnology/dGenhancer.xlsx

### dGoligo synthesis

Sense-, antisense- or microRNA-based DNA oligonucleotides were designed (S3 Table) to target *cis*-acting elements of TRβ1 5’UTRs (S1 Appendix). For *in vivo* studies nuclease-resistant 2'-O-methyl modified RNA oligonucleotides were synthesized. Oligonucleotides were performed with ABI 3900 High-Throughput DNA Synthesizer (Applied Biosystems, Foster City, CA) using standard DNA or 2'-O-methyl-modified phosphoramidites (Link Technologies, Lanarkshire, UK).

### Coupled *in vitro* transcription and translation assay

500ng of each plasmid were simultaneously transcribed and translated in 0.2mL-PCR tubes using RTS 100 Wheat Germ CECF Kit (Roche Diagnostics, Mannheim, Germany). The translation assay was conducted in 20μL of Reaction Solution, supplemented with 20uL of Feeding Solution after initial 3h-incubation. All reactions were maintained at 37°C for 6h with shaking at 600 rpm, using the RTS ProteoMaster Instrument (Roche Applied Science, Mannheim, Germany). After reaction, DNA levels of appropriate pKS plasmids (plasmid copy number per each reaction) were measured by semi-quantitative Real-Time PCR and served as internal controls of transcription and translation efficiency (S1 Appendix). mRNA levels were determined by semi-quantitative measurement of luciferase transcripts using **Real-Time PCR** (Quanti-Fast SYBR Green PCR Kit, Qiagen, Hilden, Germany) and two pairs of PCR primers (S4 Table). The reactions were performed with LightCycler 480 (Roche, Germany) under standard conditions shown in Materials and Methods in SM. *In vitro* **translation-enhancing assay** was performed with 500ng of pKS-A, pKS-F and pKS-control constructs were expressed as above in the presence of 0,25μM each tested dGoligo (S3 Table) or in the absence of dGoligo (dG-). For normalization, the results were divided by corresponding results obtained for pKS-control, to eliminate any possible non-specific dGoligo effects. Translation efficiency was determined by the use of **Luciferase Reporter Gene Assay** (Promega, Madison, WI) with the Synergy2 luminometer (BioTek, Winooski, VT) in conditions recommended by the manufacturers.

### Cell-culture based, *in vivo* transcription and translation assay

The human clear cell renal carcinoma cell line (Caki-2) was used (American Type Culture Collection, Manassas, VA). Caki-2 cells were grown in McCoy’s 5A medium with L-glutamine (Gibco/Invitrogen, Carlsbad, Ca) with 10% fetal bovine serum (FBS; Sigma-Aldrich, Saint Louis, MO) and 1x penicillin-streptomycin solution (Sigma-Aldrich, Saint Louis, MO). The cells were maintained at 37°C in 5% CO_2_ atmosphere. For all the experiments, Caki-2 cells were seeded into 75cm^2^ bottles, 6- or 12-well culture plates at density 13x10^3^ cells/cm^2^, 24h before transfection. Three independent *in vivo* experiments were performed in triplicate.

### Luciferase expressing plasmids and dGoligo transfection

24 hours after seeding, cells were transfected with 100 ng of control pRL-TK (Promega, Madison, WI) and 1ug of pGL3-A plasmid [22], using 1μg/ul PEI (Linear Polyethylenimine, Polysciences Inc., Warrington, PA) and 150mM NaCl in FBS-free McCoy’s medium. Five hours after transfection, the medium was replaced with McCoy’s medium plus 10% FBS. PEI-mediated transfection reactions contained 36nM of each dG and was carried overnight. The medium was then replaced with McCoy’s medium plus 10% FBS and 1x penicillin-streptomycin solution. 24h after the last medium replacement, cells were harvested. The cells were divided into two equal parts for isolation of total RNA and luciferase protein. The RNA was processed as described below. **Dual-luciferase assay**. The protein measurements were performed using dual-luciferase assay (Promega, Madison, WI) in the Synergy2 luminometer (BioTek, Winooski, VT), according to the manufacturer’s instructions.

### Cellular RNA isolation

Total RNA for real-time PCR was purified from the second part of the collected cells as it was described for *in vitro* assay.

### Reverse transcription and Semi-Quantitative Real-time PCR

Reverse transcription and Real-time PCR of luciferase pGL3-A and pRL-TK control was performed according to the protocol used for *in vitro* study. The transcript levels of Firefly luciferase were compared with *Renilla* using specific primers (S4 Table). Relative changes in gene expression were calculated using the 2^(-ΔΔCt)^.

### dGoligo controls

All dG*s* were tested as complementary sense and antisense sequences (S3 Table). dG5, dG6, dG7 and dG8 were synthesized as mismatched controls containing a 3 nucleotide-long mismatched insertion in the middle of the oligonucleotides (Fig 2). An additional scrambled control oligonucleotide (dGsc) with an irrelevant (random) sequence was as designed with GeneScript software (S3 Table).

### Bioinformatic analysis

Total Gibbs energy prediction (*ΔG* = ΔH—TΔS) of 5’UTR secondary structures was performed using RNAstructure version 5.2 [37]. NCBI-BLASTN program and IRESite database [38] were used for comparative sequence analysis towards evolutionary conserved 5’UTR domains such as IRES consensus sequences. The dGenhancer calculator was used to determine translation regulating elements (S1 Appendix).

### Statistics

At least three independent experiments were carried out for each assay and measured in triplicate. Normality of data distribution was estimated using Shapiro-Wilk test and in each case data were analyzed by ANOVA followed by Dunnett’s multiple comparison test. p<0.001 was considered statistically significant. Correlation of Gibbs energy and translation efficiency (Fig 1c) was estimated by r-squared value of the Pearson product-moment correlation coefficient. Logarithmically transformed data of translation efficiency were analyzed with the Gibbs energies by linear regression. p<0.05 was considered statistically significant.

## Supporting Information

S1 Appendix Supporting Materials and Methods. (PDF)

S1 Fig Folding of TRβ1 5’UTRs. (PDF)

S2 Fig Translation-enhancing, IRES-like element in TRβ1 5’UTR. (PDF)

S3 Fig Time-course of protein synthesis rates in RTS 100 Wheat Germ CECF system. (PDF)

S4 Fig Proposed folding patterns of TRβ1 5’UTR after dGoligo supplementation. (PDF)

S5 Fig Change in translation efficiency after dG1 and dG4 supplementation. (PDF)

S6 Fig Potential hsa-miR-211 target sites within TRβ1 3’UTR and 5’UTR. (PDF)

S7 Fig dGoligo binding capacity. (PDF)

S8 Fig Binding selectivity confirmed by dGoligo-primed reverse transcription. (PDF)

S9 Fig DNA-based dGoligo-mediated effects in Caki-2 cells. (PDF)

S1 Table Basic characteristics of selected TRβ1 5’UTR variants A-G. (PDF)

S2 Table Prediction of translational regulatory potential of 5’UTRs. (PDF)

S3 Table List of dGoligos (dGs) used in the study. (PDF)

S4 Table List of primers used in Real-Time and classic PCR. (PDF)

## References

[1] Van Der Kelen K, Beyaert R, Inzé D, De Veylder L. Translational control of eukaryotic gene expression. Crit Rev Biochem Mol Biol. 2009 Jul-Aug;44(4):143–68. 10.1080/10409230902882090 19604130

[2] Myasnikov AG, Simonetti A, Marzi S, Klaholz BP. Structure-function insights into prokaryotic and eukaryotic translation initiation. Curr Opin Struct Biol. 2009 6;19(3):300–9. 10.1016/j.sbi.2009.04.010 19493673

[3] Mahen EM, Watson PY, Cottrell JW, Fedor MJ. mRNA secondary structures fold sequentially but exchange rapidly in vivo. PLoS Biol. 2010 2 9;8(2):e1000307 10.1371/journal.pbio.1000307 20161716 PMC2817708

[4] Vattem KM, Wek RC. Reinitiation involving upstream ORFs regulates ATF4 mRNA translation in mammalian cells. Proc Natl Acad Sci U S A. 2004 8 3;101(31):11269–74. 15277680 10.1073/pnas.0400541101 PMC509193

[5] Wellensiek BP, Larsen AC, Stephens B, Kukurba K, Waern K, Briones N, et al Genome-wide profiling of human cap-independent translation-enhancing elements. Nat Methods. 2013 8;10(8):747–50. 10.1038/nmeth.2522 23770754 PMC3731418

[6] Chatterjee S, Pal JK. Role of 5'- and 3'-untranslated regions of mRNAs in human diseases. Biol Cell. 2009 5;101(5):251–62. 10.1042/BC20080104 19275763

[7] Loreni F, Mancino M, Biffo S. Translation factors and ribosomal proteins control tumor onset and progression: how? Oncogene. 2014 4 24;33(17):2145–56. 10.1038/onc.2013.153 23644661

[8] Boguslawska J, Wojcicka A, Piekielko-Witkowska A, Master A, Nauman A. MiR-224 targets the 3'UTR of type 1 5'-iodothyronine deiodinase possibly contributing to tissue hypothyroidism in renal cancer. PLoS One. 2011;6(9):e24541 10.1371/journal.pone.0024541 21912701 PMC3166326

[9] Kenski DM, Butora G, Willingham AT, Cooper AJ, Fu W, et al siRNA-optimized Modifications for Enhanced In Vivo Activity. Mol Ther Nucleic Acids. 2012 1 24;1:e5 10.1038/mtna.2011.4 23344622 PMC3381598

[10] Sud R, Geller ET, Schellenberg GD. Antisense-mediated Exon Skipping Decreases Tau Protein Expression: A Potential Therapy for Tauopathies. Mol Ther Nucleic Acids. 2014 10 21;3:e204 10.1038/mtna.2014.55 25072694 PMC4121519

[11] Richter JD, Sonenberg N. Regulation of cap-dependent translation by eIF4E inhibitory proteins. Nature. 2005 2 3;433(7025):477–80. 15690031 10.1038/nature03205

[12] Komar AA, Hatzoglou M. Cellular IRES-mediated translation: the war of ITAFs in pathophysiological states. Cell Cycle. 2011 1 15;10(2):229–40. 21220943 10.4161/cc.10.2.14472 PMC3048795

[13] Master A, Nauman A. Molecular mechanisms of protein biosynthesis initiation—biochemical and biomedical implications of a new model of translation enhanced by the RNA hypoxia response element (rHRE). Postepy Biochem. 2014; 60(1):39–54. 25033541

[14] Pyronnet S, Dostie J, Sonenberg N. Suppression of cap-dependent translation in mitosis. Genes Dev. 2001 8 15;15(16):2083–93. 11511540 10.1101/gad.889201 PMC312759

[15] Clemens MJ. Translational regulation in cell stress and apoptosis. Roles of the eIF4E binding proteins. J Cell Mol Med. 2001 Jul-Sep;5(3):221–39. 12067482 10.1111/j.1582-4934.2001.tb00157.x PMC6514779

[16] Van den Beucken T, Koritzinsky M, Wouters BG. Translational control of gene expression during hypoxia. Cancer Biol Ther. 2006 7;5(7):749–55. Epub 2006 Jul 1. 16861930 10.4161/cbt.5.7.2972

[17] Biro JC. Correlation between nucleotide composition and folding energy of coding sequences with special attention to wobble bases. Theor Biol Med Model.2008 7 29;5:14 10.1186/1742-4682-5-14 18664268 PMC2515297

[18] Meijer HA, Kong YW, Lu WT, Wilczynska A, Spriggs RV, Robinson SW, et al Translational repression and eIF4A2 activity are critical for microRNA-mediated gene regulation. Science. 2013 4 5;340(6128):82–5. 10.1126/science.1231197 23559250

[19] Master A, Nauman A. THRB (Thyroid Hormone Receptor, Beta). Atlas Genet Cytogenet Oncol Haematol. 2014; 18(6):400–433.

[20] Martínez-Iglesias O, Garcia-Silva S, Tenbaum SP, Regadera J, Larcher F, Paramio JM, et al Thyroid hormone receptor beta1 acts as a potent suppressor of tumor invasiveness and metastasis, Cancer Res. 69 (2009) 501–509. 10.1158/0008-5472.CAN-08-2198 19147563

[21] Bisio A, Latorre E, Andreotti V, Bressac-de Paillerets B, Harland M, Scarra GB, et al The 5'-untranslated region of p16INK4a melanoma tumor suppressor acts as a cellular IRES, controlling mRNA translation under hypoxia through YBX1 binding. Oncotarget. 2015 11 24;6(37):39980–94. 10.18632/oncotarget.5387 26498684 PMC4741874

[22] Frankton S, Harvey CB, Gleason LM, Fadel A, Williams GR. Multiple messenger ribonucleic acid variants regulate cell-specific expression of human thyroid hormone receptor beta1. Mol Endocrinol. 2004 7;18(7):1631–42. 15105435 10.1210/me.2003-0346

[23] Master A, Wojcicka A, Piekielko-Witkowska A, Boguslawska J, Poplawski P, Tanski Z, et al Untranslated regions of thyroid hormone receptor beta 1 mRNA are impaired in human clear cell renal cell carcinoma. Biochim Biophys Acta. 2010 11;1802(11):995–1005. 10.1016/j.bbadis.2010.07.025 20691260

[24] Vasudevan S, Tong Y, Steitz JA. Switching from repression to activation: microRNAs can up-regulate translation. Science. 2007 12 21;318(5858):1931–4. 18048652 10.1126/science.1149460

[25] Lee I, Ajay SS, Yook JI, Kim HS, Hong SH, Kim NH, et al New class of microRNA targets containing simultaneous 5'-UTR and 3'-UTR interaction sites. Genome Res. 2009 7;19(7):1175–83. 10.1101/gr.089367.108 19336450 PMC2704433

[26] Henke JI, Goergen D, Zheng J, Song Y, Schüttler CG, Fehr C, et al microRNA-122 stimulates translation of hepatitis C virus RNA. EMBO J. 2008 12 17;27(24):3300–10. 10.1038/emboj.2008.244 19020517 PMC2586803

[27] Birmingham A, Selfors LM, Forster T, Wrobel D, Kennedy CJ, Shanks E, et al Statistical methods for analysis of high-throughput RNA interference screens. Nat Methods. 2009 8;6(8):569–75. 10.1038/nmeth.1351 19644458 PMC2789971

[28] Jayaswal V, Lutherborrow M, Ma DD, Yang YH. Identification of microRNA-mRNA modules using microarray data. BMC Genomics. 2011 3 6;12:138 10.1186/1471-2164-12-138 21375780 PMC3065435

[29] Li LC, Okino ST, Zhao H, Pookot D, Place RF, Urakami S, et al Small dsRNAs induce transcriptional activation in human cells. Proc Natl Acad Sci U S A. 2006 11 14;103(46):17337–42. 17085592 10.1073/pnas.0607015103 PMC1859931

[30] Huang V, Qin Y, Wang J, Wang X, Place RF, Lin G, et al RNAa is conserved in mammalian cells. PLoS One. 2010 1 22;5(1):e8848 10.1371/journal.pone.0008848 20107511 PMC2809750

[31] Schwartz JC, Younger ST, Nguyen NB, Hardy DB, Monia BP, Corey DR, et al Antisense transcripts are targets for activating small RNAs. Nat Struct Mol Biol. 2008 8;15(8):842–8. 10.1038/nsmb.1444 18604220 PMC2574822

[32] Master A, Nauman A. Gene expression regulation by long naturally occurring antisense transcripts. Post. Biol. Kom. 2014;41(1):3–28.

[33] Schwartz JC, Younger ST, Nguyen NB, Hardy DB, Monia BP, Corey DR, Janowski BA. Antisense transcripts are targets for activating small RNAs. Nat Struct Mol Biol. 2008 8;15(8):842–8. 10.1038/nsmb.1444 18604220 PMC2574822

[34] Master A, Nauman A. Genomic context and expression regulation of nuclear thyroid hormone receptors by long naturally occurring antisense transcripts. Post. Biol. Kom. 2014; 41(1):29–58.

[35] Xia X, Holcik M. Strong eukaryotic IRESs have weak secondary structure. PLoS One. 2009;4(1):e4136 10.1371/journal.pone.0004136 19125192 PMC2607549

[36] Bugaut A, Balasubramanian S. 5'-UTR RNA G-quadruplexes: translation regulation and targeting. Nucleic Acids Res. 2012 6;40(11):4727–41. 10.1093/nar/gks068 22351747 PMC3367173

[37] Reuter JS, Mathews DH. RNAstructure: software for RNA secondary structure prediction and analysis. BMC Bioinformatics. 2010 3 15;11:129 10.1186/1471-2105-11-129 20230624 PMC2984261

[38] Mokrejs M, Masek T, Vopálensky V, Hlubucek P, Delbos P, et al IRESite—a tool for the examination of viral and cellular internal ribosome entry sites. Nucleic Acids Res. 2010 1;38(Database issue):D131–6. 10.1093/nar/gkp981 19917642 PMC2808886

[39] Rhoades MW, Reinhart BJ, Lim LP, Burge CB, Bartel B, Bartel DP. Prediction of plant microRNA targets. Cell. 2002 8 23;110(4):513–20. 12202040 10.1016/s0092-8674(02)00863-2

[40] Wu H, Lima WF, Zhang H, Fan A, Sun H, Crooke ST. Determination of the role of the human RNase H1 in the pharmacology of DNA-like antisense drugs. J Biol Chem. 2004 4 23;279(17):17181–9. 14960586 10.1074/jbc.M311683200

[41] Schneider PN, Olthoff JT, Matthews AJ, Houston DW. Use of fully modified 2'-O-methyl antisense oligos for loss-of-function studies in vertebrate embryos. Genesis. 2011 3;49(3):117–23. 10.1002/dvg.20689 21442720 PMC3121920

[42] Kim WG, Zhu X, Kim DW, Zhang L, Kebebew E, Cheng SY. Reactivation of the silenced thyroid hormone receptor β gene expression delays thyroid tumor progression. Endocrinology. 2013 1;154(1):25–35. 10.1210/en.2012-1728 23183175 PMC3529371

[43] Huret JL, Ahmad M, Arsaban M, Bernheim A, Cigna J, Desangles F, et al Atlas of genetics and cytogenetics in oncology and haematology in 2013. Nucleic Acids Res. 2013 1;41(Database issue):D920–4. 10.1093/nar/gks1082 23161685 PMC3531131

[44] Kim WG, Zhao L, Kim DW, Willingham MC, Cheng SY. Inhibition of tumorigenesis by the thyroid hormone receptor β in xenograft models. Thyroid. 2014 2;24(2):260–9. 10.1089/thy.2013.0054 23731250 PMC3926148

[45] Ørom UA, Nielsen FC, Lund AH. MicroRNA-10a binds the 5'UTR of ribosomal protein mRNAs and enhances their translation. Mol Cell. 2008 5 23;30(4):460–71. 10.1016/j.molcel.2008.05.001 18498749

[46] Morris KV, Santoso S, Turner AM, Pastori C, Hawkins PG. Bidirectional transcription directs both transcriptional gene activation and suppression in human cells. PLoS Genet. 2008 11;4(11):e1000258 10.1371/journal.pgen.1000258 19008947 PMC2576438

[47] Master A, Wojcicka A, Nauman A. The 5’UTR-dependent enhancement of protein translation efficiency triggered by self-transfecting 3’-aminoallyl-containing oligonucleotides (aa-dGoligos) targeting a pool of strongly folded transcript variants of the THRB suppressor gene. Mol Ther. 2014 5; 22: S31–S38;

[48] Wu H, Lima WF, Zhang H, Fan A, Sun H, Crooke ST. Determination of the role of the human RNase H1 in the pharmacology of DNA-like antisense drugs. J Biol Chem. 2004 4 23;279(17):17181–9. 14960586 10.1074/jbc.M311683200

[49] Cazenave C, Frank P, Büsen W. Characterization of ribonuclease H activities present in two cell-free protein synthesizing systems, the wheat germ extract and the rabbit reticulocyte lysate. Biochimie. 1993;75(1–2):113–22. 8389210 10.1016/0300-9084(93)90032-n

