# Supplementary material for: A Novel Method for Gene-Specific Enhancement of Protein Translation by Targeting 5’UTRs of Selected Tumor Suppressors
Source: PLoS One. 2016 May 12;11(5):e0155359. doi: 10.1371/journal.pone.0155359 (PMC4865139; doi:10.1371/journal.pone.0155359)
Supplement: S1 Appendix — (PDF) [file pone.0155359.s001.pdf]

Supporting Information

Appendix S1. Supporting Materials and Methods.

Prediction of translation-regulating elements. An excel-based calculator - dG*enhancer* was used to search for putative 5’UTR *cis*-acting elements, which functional activity could be determined by *Gibbs energy-dependent secondary structure formation*. Prediction of total Gibbs energies (ΔG=ΔH–TΔS) of the 5’UTR structures was performed using *RNAstructure* version 5.2 [s1]. These ΔGs were treated as input data for dG*enhancer* calculations showing the strongest translation-regulating signal (high peak) at nucleotides 130 and 133 located in the middle of exon 2a of TRβ1 5’UTRs (see print screens below).

All annotations and formulae are included in the calculator available under the following link: http://www.serwer1448847.home.pl/biotechnology/dGenhancer.xlsx

The dG*enhancer* can show ΔG changes observed among 5’UTR sequences containing *virtual* SNPs (red) that were substituted base by base *in silico* in each nucleotide position of the 5’UTRs, as it is shown below for two exemplary 5'UTR bases (green).

1 2 3 4 5 6 7 8 9 ... <--- nucleotide positions (nt_s) within a fragment sequence of TRβ1 variant A
5'UTR-...**A** G A G C C C G C ...-3' ΔG=-68,30 [kcal/mol]
5'UTR-...C G A G C C C G C ...-3' ΔG=-69,30 [kcal/mol]
5'UTR-...T G A G C C C G C ...-3' ΔG=-66,30 [kcal/mol]
5'UTR-...G G A G C C C G C ...-3' ΔG=-70,30 [kcal/mol]
5'UTR-...A **G** A G C C C G C ...-3' ΔG=-71,30 [kcal/mol]
5'UTR-...A A A G C C C G C ...-3' ΔG=-70,30 [kcal/mol]
5'UTR-...A C A G C C C G C ...-3' ΔG=-70,10 [kcal/mol]
5'UTR-...A T A G C C C G C ...-3' ΔG=-65,30 [kcal/mol] ... and so on ... for all 5'UTR bases

As a result, the calculator makes a graph presenting nucleotide stretches (elements), which substitution can change the total 5'UTR Gibbs energy the most, thereby indicating regions that could be characterized by the highest potential to regulate protein synthesis (*translational regulatory potential*). Oligonucleotide-based *trans*-acting factors (termed here d*Goligos,* dGs), which are designed to selectively bind to these 5'UTR regions, could block or release their translation -silencing or -enhancing elements. As chemically synthesized siRNAs and ASOs, dGs are highly sequence-specific nucleic acid molecules, but on the contrary to the gene-silencing oligonucleotides, allow for specific binding to their target sequence followed by selective enhancement of protein synthesis.

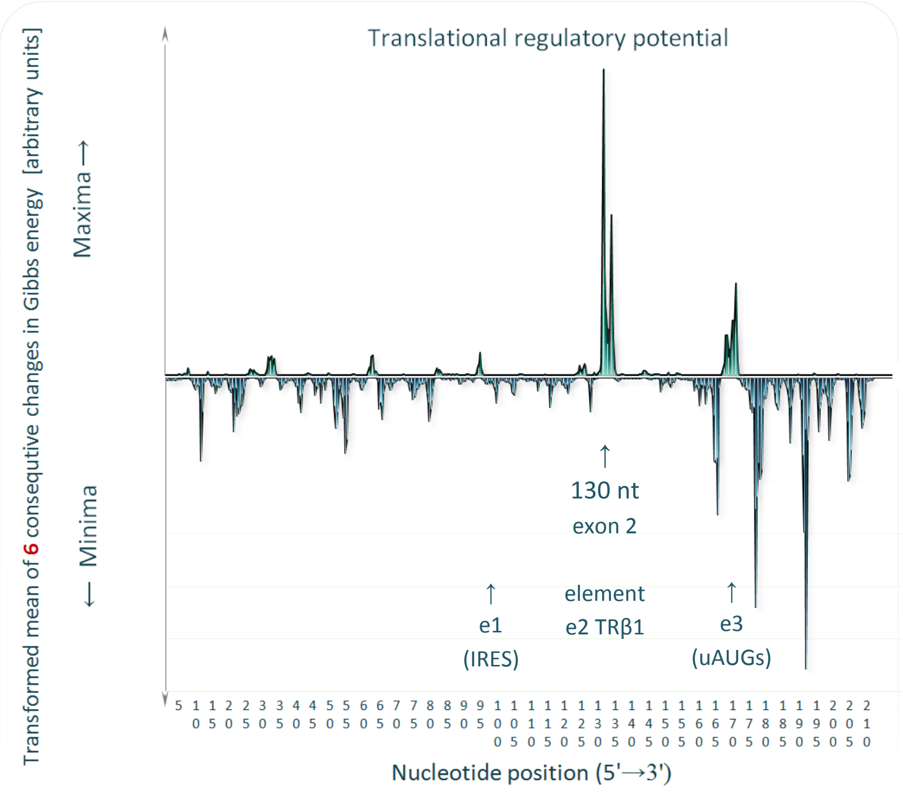

← (**a**) a graph generated by dG*enhancer.* Variant A of TRβ1 5’UTR ΔG= -67,8 [kcal/mol].

Maxima and Minima may indicate translation-regulating elements that are specifically dependent on a sequence folding state.

Maxima ~ putative translation-silencing elements that can inhibit translation in a normal folding state of a 5'UTR (...should be blocked to enhance translation).

Minima ~ putative translation-enhancing elements that can elevate translation in a normal folding state of a 5'UTR (...should be released to enhance translation).

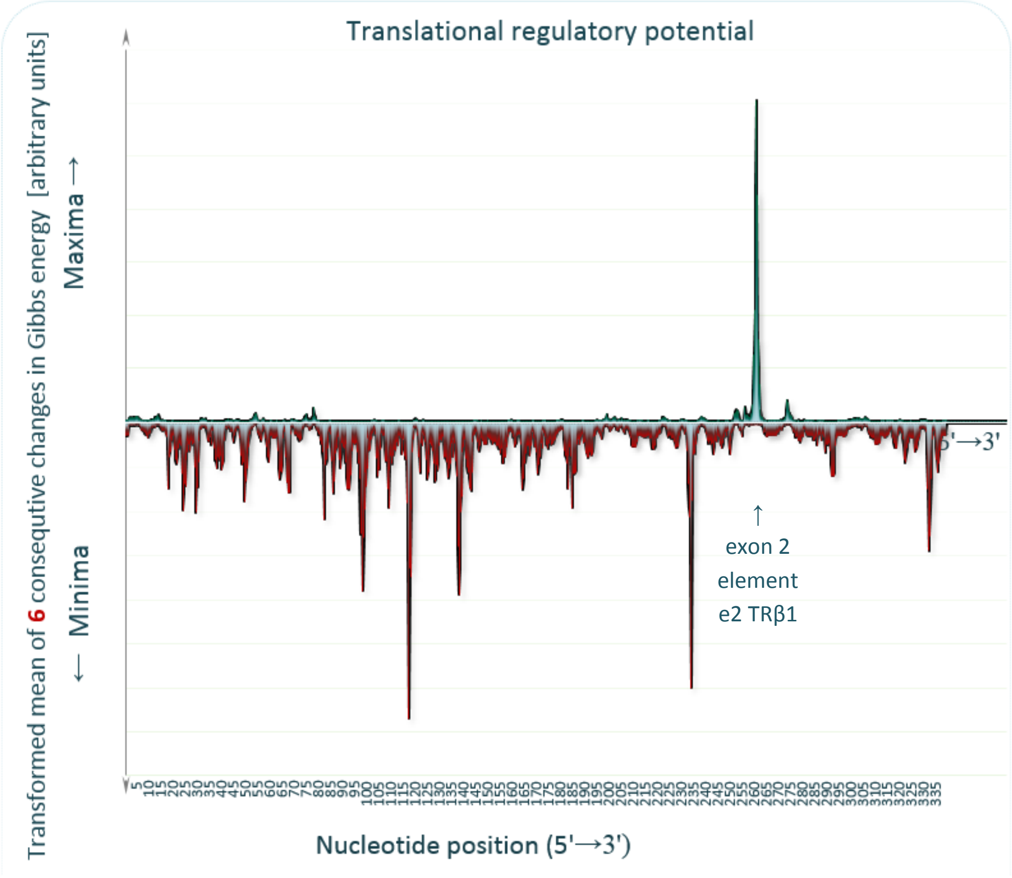

← (**b**) a graph generated by dG*enhancer.* Variant F of TRβ1 5’UTR.

ΔG= - 125,3[kcal/mol].

E = Max ΔG - n*ΔG = 6,40 [kcal/mol] ~ susceptibility to translation enhancement (more info. in section d).

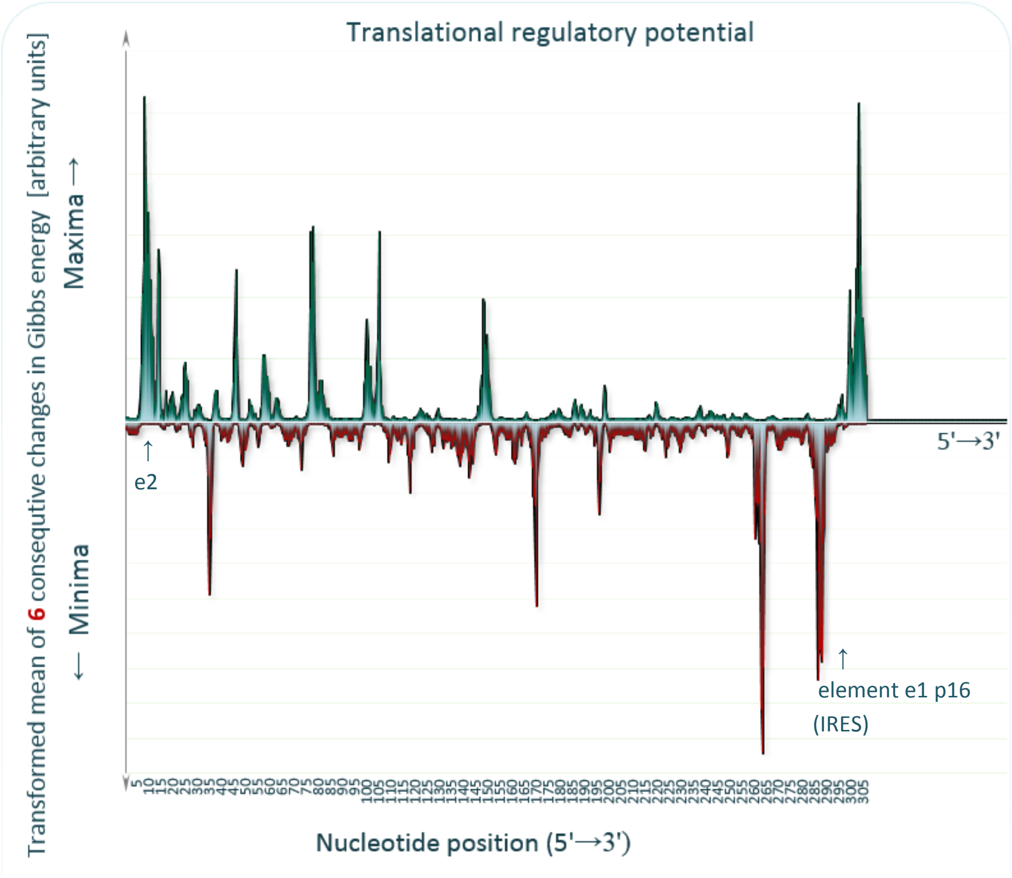

← (**c**) a graph generated by dG*enhancer.*

5’UTR of variant p16INK4a (CDKN2A).

ΔG= - 146,4 [kcal/mol],

E = Max ΔG - n*ΔG = 7,10 [kcal/mol].

(**d**) Selected print screens of dG*enhancer* calculations (Variant A of TRβ1 5'UTR).

All annotations and formulae are included in the dG*enhancer* calculator.

Put your data in the table		Tested sequence (D3)	Sequence length [nt]
Paste your sequence as a text	⇒	AAGAGCCCGCAGGCTACCTTCCCCGGG CAGGGGCGCTCAACCCAACCGGCTCCA	211
Sample name	⇒	Variant A of TRbeta1 5'UTR	
Go to [2-sort the results]	⇒	OK	

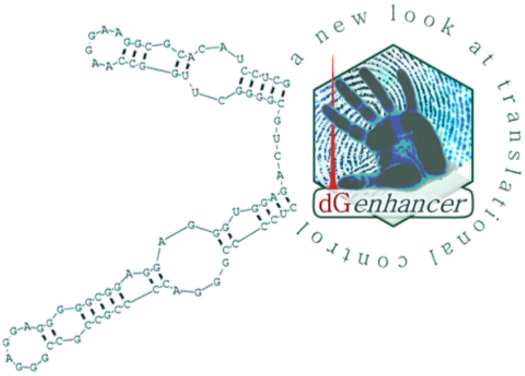

Short instruction (with internal links):

1. Paste the sequence of your interest into D3 cell. The sequence may have no gaps or other signs (only A G C and T are allowed)

2. Copy (as text) each sequence from column M and calculate value of Gibbs energy for each one (use any available software to calculate the gibbs energy), then clear old (exemplary) data in column P and paste your new data in cells of column P.

3. Sort the results (2-...) and take your results (3-...).

2016-03-26 22:41

Analysis parameters:		Reference parameters
First substituting nucleotide →	A	A, G, C, or T
Second substituting nucleotide →	C	A, G, C, or T
Third substituting nucleotide →	T	A, G, C, or T
Fourth substituting nucleotide →	G	A, G, C, or T
Number of substitutions per base →	3	3 or 4
Exponent →	140	20-200
Enhancing (E) or Silencing (S) effects →	E	E or S
Length of designed dGoligo →	21	any
SNP position within sense-like dGoligo →	11	any
Loop position within microRNA-like dG →	11	any
Sequence of the loop →	ttt	any

OK

Take your results

Nucleotide position ↓ ↓ Base of the tested seq. ↓ Artificial substitution P = ΔGs ↓

	5'		5'	M	P
1	A	>	A	AAGAGCCCGCAGGCTACCTTCC ...	-67,80
1	A	>	C	CAGAGCCCGCAGGCTACCTTCC ...	-67,80
1	A	>	T	TAGAGCCCGCAGGCTACCTTCC ...	-67,80
1	A	>	G	GAGAGCCCGCAGGCTACCTTCC ...	-67,80
2	A	>	C	ACGAGCCCGCAGGCTACCTTCC ...	-67,80
2	A	>	T	ATGAGCCCGCAGGCTACCTTCC ...	-67,80
2	A	>	G	AGGAGCCCGCAGGCTACCTTCC ...	-69,70
3	G	>	A	AAAAGCCCGCAGGCTACCTTCC ...	-67,80
3	G	>	C	AACAGCCCGCAGGCTACCTTCC ...	-67,80
3	G	>	T	AATAGCCCGCAGGCTACCTTCC ...	-68,00
4	A	>	C	AAGCGCCCGCAGGCTACCTTCC ...	-67,50
4	A	>	T	AAGTGCCCGCAGGCTACCTTCC ...	-68,40
4	A	>	G	AAGGGCCCGCAGGCTACCTTCC ...	-68,40
17	C	>	G	AAGAGCCCGCAGGCTAGCTTCC ...	-68,50
17	C	>	A	AAGAGCCCGCAGGCTAACTTCC ...	-66,30
18	C	>	T	AAGAGCCCGCAGGCTACTTTCC ...	-67,50
18	C	>	G	AAGAGCCCGCAGGCTACGTTCC ...	-68,00
18	C	>	A	AAGAGCCCGCAGGCTACATTCC ...	-68,10
19	T	>	G	AAGAGCCCGCAGGCTACCGTCC ...	-69,40
19	T	>	A	AAGAGCCCGCAGGCTACCATCC ...	-66,40
19	T	>	C	AAGAGCCCGCAGGCTACCCTCC ...	-70,20
20	T	>	G	AAGAGCCCGCAGGCTACCTGCC ...	-71,40
20	T	>	A	AAGAGCCCGCAGGCTACCTACC ...	-67,80
20	T	>	C	AAGAGCCCGCAGGCTACCTCCC ...	-68,80
21	C	>	T	AAGAGCCCGCAGGCTACCTTTC ...	-68,50
21	C	>	G	AAGAGCCCGCAGGCTACCTTGC ...	-71,20
21	C	>	A	AAGAGCCCGCAGGCTACCTTAC ...	-67,80
22	C	>	T	AAGAGCCCGCAGGCTACCTTCT ...	-68,80
23	C	>	T	AAGAGCCCGCAGGCTACCTTCC ...	-69,10
23	C	>	G	AAGAGCCCGCAGGCTACCTTCC ...	-70,40
23	C	>	A	AAGAGCCCGCAGGCTACCTTCC ...	-66,10
24	C	>	T	AAGAGCCCGCAGGCTACCTTCC ...	-70,60
24	C	>	G	AAGAGCCCGCAGGCTACCTTCC ...	-69,10
24	C	>	A	AAGAGCCCGCAGGCTACCTTCC ...	-65,50
25	G	>	A	AAGAGCCCGCAGGCTACCTTCC ...	-64,90
25	G	>	C	AAGAGCCCGCAGGCTACCTTCC ...	-69,10
25	G	>	T	AAGAGCCCGCAGGCTACCTTCC ...	-66,90
26	G	>	A	AAGAGCCCGCAGGCTACCTTCC ...	-65,80
26	G	>	C	AAGAGCCCGCAGGCTACCTTCC ...	-68,40
26	G	>	T	AAGAGCCCGCAGGCTACCTTCC ...	-66,50
27	G	>	A	AAGAGCCCGCAGGCTACCTTCC ...	-66,60
27	G	>	C	AAGAGCCCGCAGGCTACCTTCC ...	-66,60
27	G	>	T	AAGAGCCCGCAGGCTACCTTCC ...	-68,40
28	C	>	T	AAGAGCCCGCAGGCTACCTTCC ...	-67,80
28	C	>	G	AAGAGCCCGCAGGCTACCTTCC ...	-68,00
28	C	>	A	AAGAGCCCGCAGGCTACCTTCC ...	-67,80
29	A	>	C	AAGAGCCCGCAGGCTACCTTCC ...	-71,20
29	A	>	T	AAGAGCCCGCAGGCTACCTTCC ...	-69,40
29	A	>	G	AAGAGCCCGCAGGCTACCTTCC ...	-68,30
30	G	>	A	AAGAGCCCGCAGGCTACCTTCC ...	-65,10
30	G	>	C	AAGAGCCCGCAGGCTACCTTCC ...	-67,40
30	G	>	T	AAGAGCCCGCAGGCTACCTTCC ...	-66,40

Sequences of the most importance

Sense dGoligo covering SNP (in the middle)	Sense dGoligo covering SNP (in the middle)	Sense, microRNA-like dGoligo containing a loop located in SNP	Nucleotide position
Maximum ΔG [kcal/mol]:	-63,5		
Forward:	GGCTGTCCTGCGTGGGTGCCA	GGCTGTCCTGCtttGTGGGTGCCA	130
Reversed:	TGGCACCCACGCAGGACAGCC	TGGCACCCACAAAGCAGGACAGCC	130
Minimum ΔG [kcal/mol]:	-69,1		
Forward:	AGTTCCACACATGATTTAATG	AGTTCCACACAtttTGATTTAATG	151
Reversed:	CATTAAATCATGTGTGGAACT	CATTAAATCAAAATGTGTGGAACT	151
n*ΔG of "native" seq. [kcal/mol]:	-67,80		

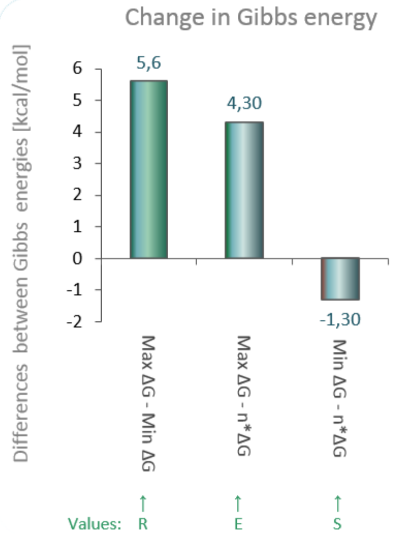

Max ΔG = max. value of Gibbs energy.
Maximum of Gibbs energy after substitution of a nucleotide (virtual SNP). The least folded sequence.
Less negative value.
~ the state that can facilite the translation initiation.

Min ΔG = min. value of Gibbs energy.
Minimum of Gibbs energy after substitution of a nucleotide (virtual SNP). The most strongly folded sequence.
More negative value.
~ the state that can inhibit the translation initiation.

n*ΔG
Value of Gibbs energy of non-substituted, native sequence.

G = Max ΔG - Min ΔG
~ susceptibility to ΔG-dependent translation regulation.

E = Max ΔG - n*ΔG ≈ how much the SNP could enhance translation efficiency,
~ susceptibility to dG-mediated translation enhancement.

S = Min ΔG - n*ΔG ≈ how much the SNP could silence translation efficiency
~ susceptibility to dG-mediated translation silencing.

dG*oligo* design and synthesis. dGs were synthesized as a structurally diverse group of sense-, antisense- or microRNA-like DNA oligonucleotides (Table S3). dGs were designed to target the most stable (showing the most negative ΔG) secondary structures of indicated *cis*-acting elements of TRβ1 5’UTRs, thus the primary function of synthetic dGs was to change the *Gibbs energy-dependent secondary structure formation* [s2, s3]. Except for a short 3-nt loop structure in microRNA-like dGs (dG5, dG6, dG9, dG10), the oligonucleotides share full homology with human TRβ1 mRNA sequence (NCBI GeneBank Acc. No. NM_000461), 5’UTR variant A (GeneBank Acc. No. AY286465.1) and 5’UTR variant F (GeneBank Acc. No. AY286470.1). dGs were expected to target one of the sequences: a) element e1 containing a putative IRES site (Master et al. 2010) located on exon 1c/2a junction (dG1, 2, 5, 6), b) element e3 - a sequence conserved among all TRβ1 5’UTR variants, containing multiple alternative AUGs (Fig 2), located on exon 2a/3 junction (dG3, 4) or c) a target site detected automatically with dG*enhancer* calculator in the middle of exon 2 (dG7, 8, 9 and 10). All dG*s* were designed as pairs of a) antisense strand (dG2, 4, 6, 8, 10) directly recognizing the indicated regulatory sequence (IRES, uAUG or dG*enhancer*-detected translation regulating element) on the TRβ1 5’UTR and b) sense strand (dG1, 3, 5, 7, 9) releasing the indicated region by binding to a sequence that folds with these regions. (Table S3, Fig 2). All oligonucleotides were synthesized on ABI 3900 High-Throughput DNA Synthesizer (Applied Biosystems, Foster City, CA) using standard DNA phosphoramidites or 2’-O-methyl modified RNA phosphoramidites (Link Technologies, Lanarkshire, UK), deprotected by treatment with a 50:50 mixture of ammonium hydroxide and aqueous methylamine (AMA) (Sigma-Aldrich, Saint Louis, MO) and purified on HPLC using Transgenomic Wave System (Transgenomic Omaha, NE).

dG*oligo* binding. The direct dG binding to RNA targets was confirmed with a standard gel-electrophoresis technique and using an approach based on primer extension by reverse transcriptase. Proper length and quality of PCR products was confirmed in agarose gel electrophoresis (Fig S7). Target RNA for dG*oligo* (dG) binding was obtained by in vitro T7 polymerase-mediated transcription of pKS-A or pKS-F plasmids. Before electrophoresis, RNA (containing TRβ1 5’UTR A or F and downstream coding sequence of luciferase) was treated with DNase I (Fermentas, Vilnius, Lithuania) to remove remnant plasmid DNA and purified with RNeasy MinElute Cleanup Kit (Qiagen, Hilden, Germany). Then, 80ng RNA was denatured, co-hybridized with 20pmol of a single dG and stained with SYBR Green I (Fig S7). **Binding selectivity** of dGs was assessed by measuring their ability to drive synthesis of specific cDNAs during reaction of reverse transcription, wherein each tested dG served as a specific primer for reverse transcriptase that requires complementarity between a target sequence and, at least, 3’-end of an oligonucleotide (Fig S8). pKS-A and pKS-F transcripts served as a template for DNA-based antisense-like dGs. Sense-like dGs share the same sequence with matrix RNA, thus were expected to have no effects on transcription of the RNA. In case of the sense dGs, instead of RNA, we used purified first strand cDNA as a template. dG-primed products were synthesized by reverse transcription of pKSs’ RNA with RevertAidTM H Minus First Strand cDNA Synthesis Kit (Fermentas, Vilnius, Lithuania). The RNAs were previously treated with DNase I (Fermentas, Vilnius, Lithuania) and purified with RNeasy MinElute Cleanup Kit (Qiagen, Hilden, Germany). Then, standard PCR was performed to confirm the expected dG-primed products (Fig S8). Due to a tendency of 2'-O-methyl groups to impede reverse transcriptase [s4], binding of dGs modified by this group were tested only by the standard gel-electrophoresis (Fig S7).

Genetic constructs containing 5'UTRs. Preparation of luciferase reporter constructs containing different TRβ1 5’UTR variants is described by Francton et al [s5]. **Linear expression construct containing p16INK4a 5’UTR** was performed by assembling: T7 promoter, 5’UTR of p16INK4a (306nt) and luciferase reporter sequence with its 3’UTR. The construct was carried out using a three-step overlap extension PCR protocol [s6] that was elaborated on the basis of principles described by

Roche in the RTS Wheat Germ LinTempGenSet manual [s7, s8] (now distributed by 5 PRIME). In the first step, p16INK4a 5'UTR-specific PCR starters (SI.F and SI.R, Table S4) were used to add overlap regions to the amplified sequence of p16INK4a 5'UTR (see scheme below). T7 promoter and a 5' fragment of luciferase coding sequence (CDS) were added to the flanking primers (cSIII.F-T7.p, cSII.F). The luciferase with 3'UTR was amplified in the second step, wherein luciferase-specific primers (SII.F, SII.R) were used to add overlap regions to the luciferase CDS. Both SI.R and SII.R contained overlap regions (cSIII.F, cSIII.R) for amplifying primers used in the third step. Human cDNA and pGL3 Luciferase Reporter Vector (pGL3-control vector, Promega) were used as a template for the first and second step, respectively. In the third step, overlap extension PCR, the products of the first and second PCR annealed with the added flanking primers (SIII.F, SIII.R) and the 5' and 3' ends were extended. Due to high GC-content in 3'-end of p16INK4a 5'UTR preamplification of the 5'UTR was performed (PCR 0), using shorter primers: S0.F and S0.R, which included one degenerated base to facilitate the PCR 0 (amplicon length = 306bp). Subsequent PCR reactions were performed using the following oligonucleotides: PCR-I (367bp): SI.F(that includes: cSIII.F – T7p. – p16 5'UTR) SI.R(cSII.F); PCR-II (2234bp) SII.F, SII.R(cSIII.R); PCR-III (2234bp) SIII.F, SIII.R (Table S4). Finally, the linear expression construct (2234bp) was ready for subsequent coupled in vitro transcription-translation performed using RTS 100 Wheat Germ CECF system. This reaction was carried out in the same way as it was described in TRβ1 studies (see article). The following dGs were used: sense dG1p16, antisense dG2p16, microRNA-like sense dG3p16, microRNA-like antisense dG4p16 and scrambled control dGscp16 (Table S3). MicroRNA-like loop was created by adding two non-complementary bases in the middle of dG1p16 and dG2p16. Reverse transcription and semi-quantitative Real-Time PCR was performed as described in Materials and Methods using the same primer pairs. Luc-rev-r, T7prom-f, Luc-RT-f, Luc-RT-r (Table S4).

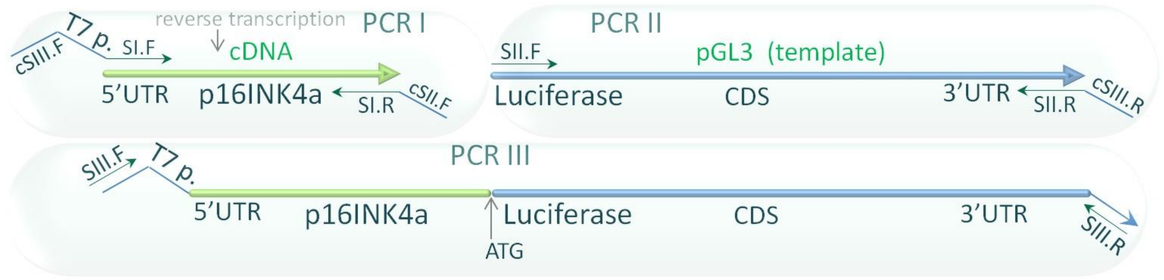

Scheme of PCR-amplified linear expression construct containing 5'UTR of p16INK4a (*CDKN2A*). This construct was generated to serve as a template in coupled *in vitro* transcription/translation assay. T7 promoter (T7.p), 5'UTR of p16INK4a (306nt), luciferase reporter sequence (CDS) together with its 3'UTR were assembled using a three-step overlap extension PCR protocol. SI.F, SI.R, SII.F, SII.R, cSII.F, cSIII.F and cSIII.R represent names of primers (Table S4) that were used in the three-step PCR (PCRI, PCRII and PCRIII). Human cDNA and pGL3 Luciferase Reporter Vector (pGL3-control vector, Promega) were used as templates for the first (PCRI) and second step (PCRII) respectively.

Analysis of translational regulatory potential of TRβ1 5'UTRs. Since the Translation Regulatory Potential (TRP) was important for predicting the 5'UTR target sites for dGs, we tried to determine a numerical parameter that could assess the TRP of our mRNA variants. To determine the TRP of TRβ1 5'UTRs we used an exemplary single nucleotide polymorphism (SNP, refID: rs62255380) relating to C219T on a putative TRβ1 IRES domain located in exon 2. This SNP was the only one polymorphism of TRβ1 5'UTR, validated in NCBI SNP database that could alter *Gibbs energy-dependent secondary structure formation* of all TRβ1 5'UTR variants. In other words, we tried to determine the translation regulatory potential of various TRβ1 5'UTRs by assesing the effects of the C219T substitution on theoretical translation efficiency (TTE). The calculations and results are shown in Table S2.

Translation-enhancing assay. This experiment was performed to assess translation-enhancing effects triggered by dG*oligos* (dGs). TRβ1 5'UTR-specific, translation-enhancing assay was designed on the basis of a previous observation that one of transcript variants encoded by C*DKN2A* suppressor gene (NCBI Gene ID: 1029) can be efficiently enhanced in the presence of a PCR sense primer directed to its strongly folded 5'UTR. Universality of this approach was confirmed by the use of TRβ1 5'UTR- and p16INK4a 5'UTR-specific dGs. 500ng of the plasmids pKS-A, pKS-F and pKS-control were transcribed and translated in the presence of 0,25μM of tested dG (Table S3) or in the absence of any dG (control), using RTS 100 Wheat Germ CECF system (Roche Diagnostics, Mannheim, Germany) in conditions described in the article. mRNA levels and luciferase activity measured in each experiment were divided by the corresponding results obtained for pKS-control lacking a TRβ1 or p16INK4a 5'-UTR. Reaction mixtures were collected for analysis by luciferase assay and real-time PCR. Reactions were performed in triplicate in three independent assays (Fig 4, Fig 5).

Translation controlled by IRES-like element in TRβ1 5'UTR. Since an alternate cap-independent, IRES-dependent translation is demonstrated to be activated by serum deprivation, which can initiate integrated stress response (ISR) [s9, s10], we performed a simple study to determine whether serum-starved Caki-2 cells (clear cell Renal Cell Cancer) can change 5'UTR-controlled translation efficiency of a downstream coding sequence. We used pGL3-A expression plasmid [s5] containing 5'UTR variant A, which has been reported to possess an IRES-like sequence located at exon 1c/2a boundary [s11]. The measurements were shown in relation to pGL3-control plasmid containing an irrelevant synthetic vector-based leader sequence lacking any TRβ1 5'UTR. Caki-2 cells were seeded at 5×105 cells per well using 12-well plates and cultured 24 hrs in McCoy's medium supplemented with 10% FBS. After 24 hrs the cells were transfected with 100ng pRL-TK and 1μg of pGL3-A or pGL3-control plasmids, using 1μg/μl PEI and 150 mM NaCl in FBS-free McCoy's medium. 5 hrs after, transfection the medium was replaced with fresh FBS-free medium to induce ISR caused by serum deprivation. At the same time, control cell cultures were supplemented with 10% FBS. Proliferation of the serum-starved Caki-2 cells but not FBS-supplemented cells was inhibited that was assessed by cell counting. The cells were maintained at 37°C in 5% CO2 atmosphere, harvested after 24 hrs and quickly divided into 2 equal parts – for isolation of total RNA and luciferase protein. Luciferase mRNA levels were assessed with Real-Time PCR and the protein measurements were performed using dual-luciferase assay in the Synergy2 luminometer. The levels of firefly luciferase activity (pGL3-A) were normalized to activity of constitutively expressed Renilla luciferase (pRL-TK). Materials used in this study are described in the article. Data from three independent experiments were performed in 12 repeats. The Shapiro–Wilk test was used to determine normality of data distribution. Normally distributed data were analyzed by ANOVA followed by Dunnett's multiple comparison test, *p< 0.01, **p<0.0001 vs. control (Fig S2).

Measurements of transcripts. Control mRNA levels were determined using quantitative real-time PCR method (Q-PCR), performed with LightCycler® 480 (Roche, Germany). Reaction mixtures of coupled transcription-translation containing equal quantity of reporter constructs were purified using GeneMATRIX Universal RNA Purification Kit (EURx, Gdansk, Poland). Reverse transcription in experiments with luciferase-containing plasmids was performed directly on the purified reaction mixture, using specific primer Luc-rev-r (Table S4, Fig 8) and the RevertAidTM H Minus First Strand cDNA Synthesis Kit (Fermentas, Vilnius, Lithuania). 1μl of 5x diluted reverse transcription reaction was used for further Q-PCR reactions using Quanti-Fast SYBR Green PCR Kit (Qiagen, Hilden, Germany) and first pair of primers: Luc-RT-f and Luc-RT-r amplifying both luciferase DNA (plasmid vector) and cDNA (RNA reverse transcription product), under the following conditions: 95°C 5min; 50 cycles: 95°C 10s, 57°C 15s, 72°C 15s; melting curve analysis: 135 cycles: 50°C; 0.3°C increase in each cycle. Ct data were acquired after reaching the threshold in real-time module, usually between 18

and 36 cycle; cycle efficiency was corrected using LightCycler® 480 (Roche, Germany). Standard curve was prepared using serial dilutions of luciferase cDNA amplification products. Second Q-PCR reaction was performed using second pair of primers: T7prom-f and Luc-RT-r (Table S4), specific only to the template vector DNA, serving as internal control for transcript levels. The final amount of each transcript was calculated by dividing quantity of the PCR products of first primer pair (amplifying both DNA and RNA) and the second primer pair (amplifying only DNA). Relative changes in gene expression were calculated using $2^{(-\Delta\Delta Ct)}$ [s12]. Levels of naturally occurring mRNAs in *in vivo* experiments were determined as described above, using transcript-specific primers (Table S4).

References for supporting materials and methods:

s1 Reuter JS, Mathews DH. RNAstructure: software for RNA secondary structure prediction and analysis. BMC Bioinformatics. 2010 Mar 15;11:129.

s2 Lu ZJ, Gloor JW, Mathews DH. Improved RNA secondary structure prediction by maximizing expected pair accuracy. RNA. 2009 Oct;15(10):1805-13.

s3 Tinoco I Jr, Collin D, Li PT. The effect of force on thermodynamics and kinetics: unfolding single RNA molecules. Biochem Soc Trans. 2004 Nov;32(Pt 5):757-60.

s4 Maden BE. Mapping 2'-O-methyl groups in ribosomal RNA. Methods. 2001 Nov;25(3):374-82.

s5 Frankton S, Harvey CB, Gleason LM, Fadel A, Williams GR. Multiple messenger ribonucleic acid variants regulate cell-specific expression of human thyroid hormone receptor beta1. Mol Endocrinol. 2004 Jul;18(7):1631-42.

s6 Swartz JR. *Cell-Free Protein Expression*. Springer Science & Business Media, New York 2012,

s7 Mc Pherson MJ, Moller SG. *PCR, The basic from background to bench*. 1st published, BIOS Scientific Publishers Ltd., Oxford, UK. 2000.

s8 Singh H, Makino S, Endo Y, Li Y, Stephens AN, Nie G. Application of the wheat-germ cell-free translation system to produce high temperature requirement A3 (HtrA3) proteases. Biotechniques. 2012 Jan;52(1):23-8.

s9 Mokrejs M, Masek T, Vopálensky V, Hlubucek P, Delbos P, Pospísek M. IRESite--a tool for the examination of viral and cellular internal ribosome entry sites. Nucleic Acids Res. 2010 Jan;38(Database issue):D131-6.

s10 Komar AA, Hatzoglou M. Cellular IRES-mediated translation: the war of ITAFs in pathophysiological states. Cell Cycle. 2011 Jan 15;10(2):229-40.

s11 Master A, Nauman A. Molecular mechanisms of protein biosynthesis initiation - biochemical and biomedical implications of a new model of translation enhanced by the RNA hypoxia response element (rHRE). Postepy Biochem. 2014; 60(1):39-54.

s12 Livak KJ, Schmittgen TD. Analysis of relative gene expression data using real-time quantitative PCR and the 2(-Delta Delta C(T)) Method. Methods. 2001 Dec; 25(4):402-8.
