# Supplementary material for: A Novel Method for Gene-Specific Enhancement of Protein Translation by Targeting 5’UTRs of Selected Tumor Suppressors
Source: PLoS One. 2016 May 12;11(5):e0155359. doi: 10.1371/journal.pone.0155359 (PMC4865139; doi:10.1371/journal.pone.0155359)
Supplement: S1 Fig — (PDF) [file pone.0155359.s002.pdf]

Supporting figures

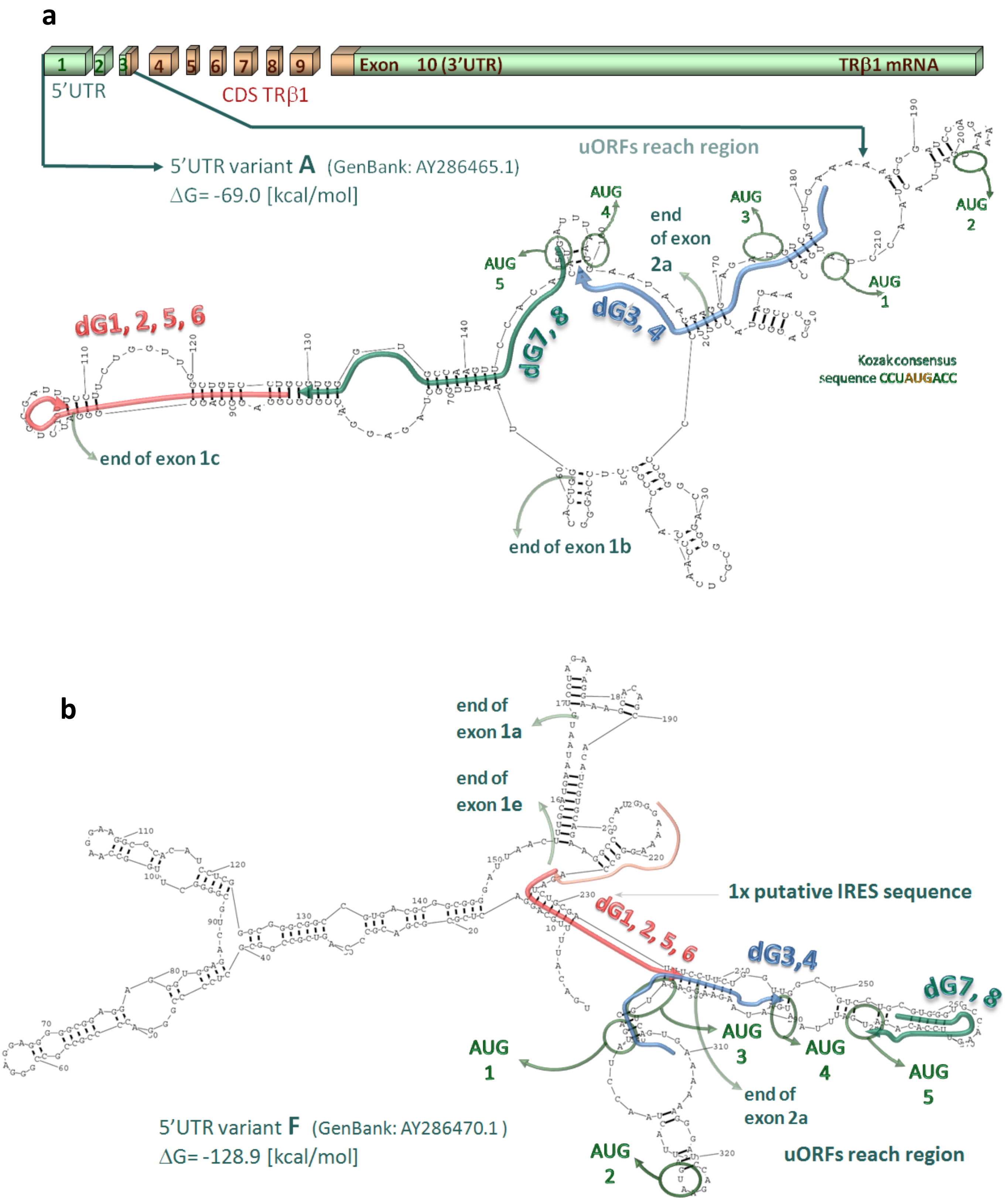

c

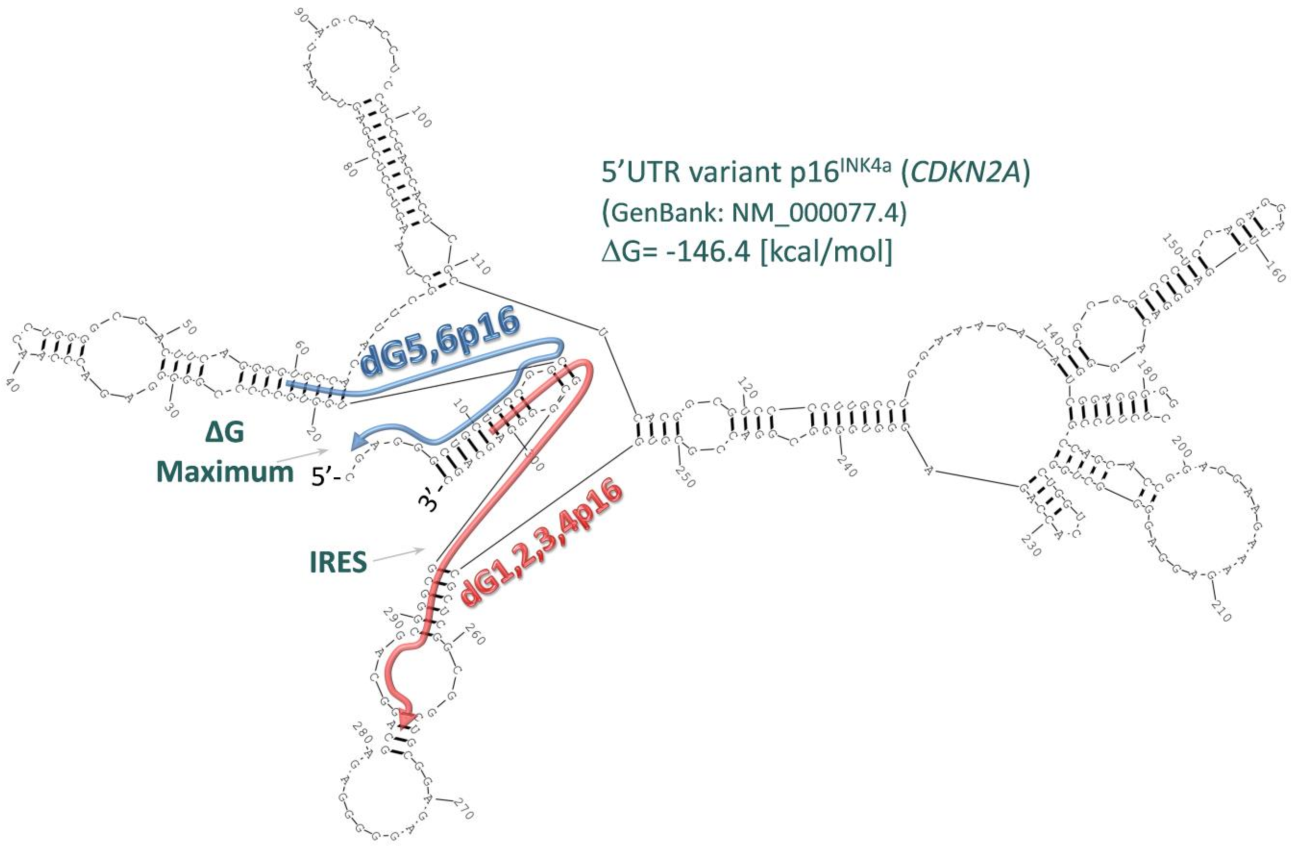

Fig S1. Folding of TRβ1 5'UTRs.

Secondary structures were modeled using RNAstructure version 5.2. (**a**) Weakly folded 5'UTR variant A (ΔG=-69.0 kcal/mol) and (**b**) Strongly folded 5'UTR variant F (ΔG=-128.9 kcal/mol) with indicated 5'UTR exons and coding sequence of TRβ1 mRNA (GeneBank Acc. No. NM_000461). Variant F lacks ex1c present in variant A what results in incomplete sequence homology at 3'-end of antisense dG2. Putative IRES sequences (Master et al. 2010), uAUGs (Frankton et al. 2004), exon-exon junctions and dG binding sites are indicated with arrows in both figures. This set of enhancing dG*s* can result in simultaneous unblocking of putative IRES sequence and blocking of uAUGs-rich region leading to significant enhancement of translation efficiency (see Fig 4). (**c**) Strongly folded 5'UTR variant p16^{INK4a} encoded (*CDKN2A*) (ΔG=-146.4 kcal/mol, GeneBank Acc. No. NM_000077.4) with indicated dGp16 target sites, ΔG maximum (identified by dG*enhancer)* and IRES sequence reported by Bisio et al. 2015.
