# Supplementary material for: A Novel Method for Gene-Specific Enhancement of Protein Translation by Targeting 5’UTRs of Selected Tumor Suppressors
Source: PLoS One. 2016 May 12;11(5):e0155359. doi: 10.1371/journal.pone.0155359 (PMC4865139; doi:10.1371/journal.pone.0155359)
Supplement: S2 Fig — (PDF) [file pone.0155359.s003.pdf]

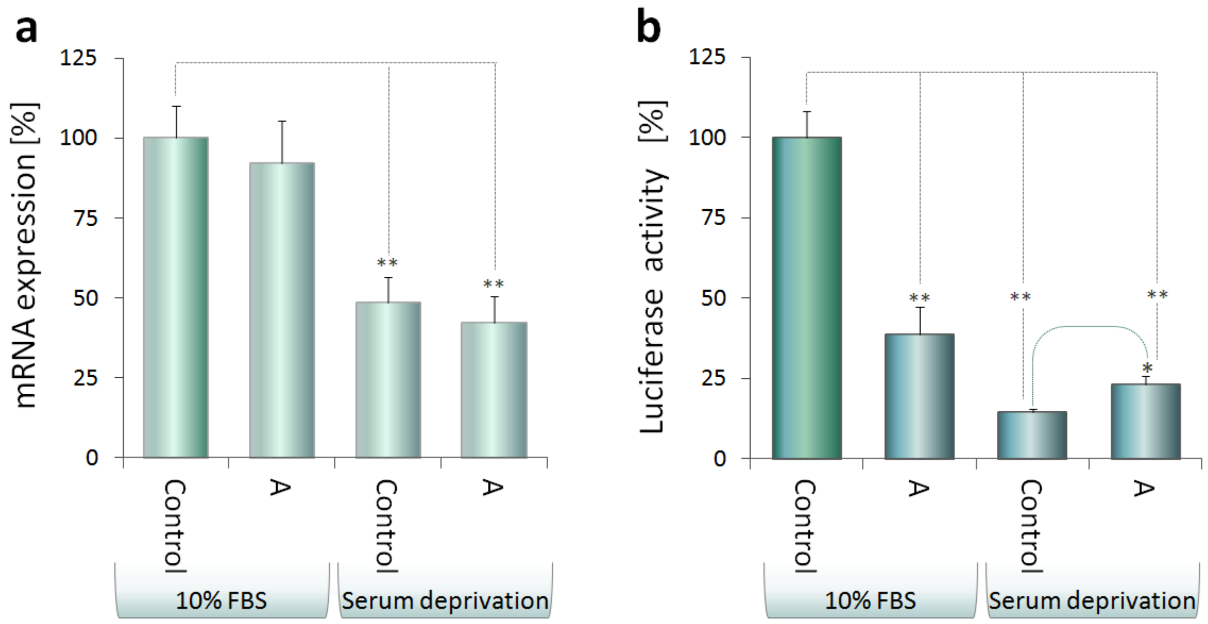

Fig S2. Translation-enhancing, IRES-like element in TRβ1 5'UTR.

This figure shows functional properties of a putative IRES-like element of TRβ1 5'UTRs, activated in response to serum starvation in Caki-2 cell culture (see Appendix S1). We used pGL3-A expression plasmid containing 5'UTR variant A (A), which has been reported to possess a putative IRES-like sequence located at exon 1c/2a boundary (see Fig 2). (**a**) Luciferase mRNA levels of pGL3-A (A) are shown relative to control plasmid - pGL3-Control (Control). Transcript levels were significantly reduced in serum-deprived Caki-2 cells (A and Control). (**b**) Luciferase activity (protein) levels of pGL3-A are shown relative to pGL3-Control. 2.59-, 6.92- and 4.36-fold lower translation rates of variant A in 10% FBS, Control and variant A in serum deprived medium were noted relative to Control in 10% FBS. Simultaneously, 1.59-fold higher luciferase protein levels were detected in serum-deprived cells transfected with pGL3-A when compared to pGL3-Control showing that the TRβ1 5'UTR contains a *cis*-acting element allowing for relatively efficient translation under serum-deprived conditions. These results are consistent with previously reported putative IRES-site in the TRβ1 5'UTR and may support our mechanistic model of dG action (Fig S4). Data from three independent experiments were performed in 12 repeats. The Shapiro–Wilk test was used to determine normality of data distribution. Normally distributed data were analyzed by ANOVA followed by Dunnett's multiple comparison test, *p< 0.01, **p<0.0001 vs. control.
