# Supplementary material for: A Novel Method for Gene-Specific Enhancement of Protein Translation by Targeting 5’UTRs of Selected Tumor Suppressors
Source: PLoS One. 2016 May 12;11(5):e0155359. doi: 10.1371/journal.pone.0155359 (PMC4865139; doi:10.1371/journal.pone.0155359)
Supplement: S3 Fig — (PDF) [file pone.0155359.s004.pdf]

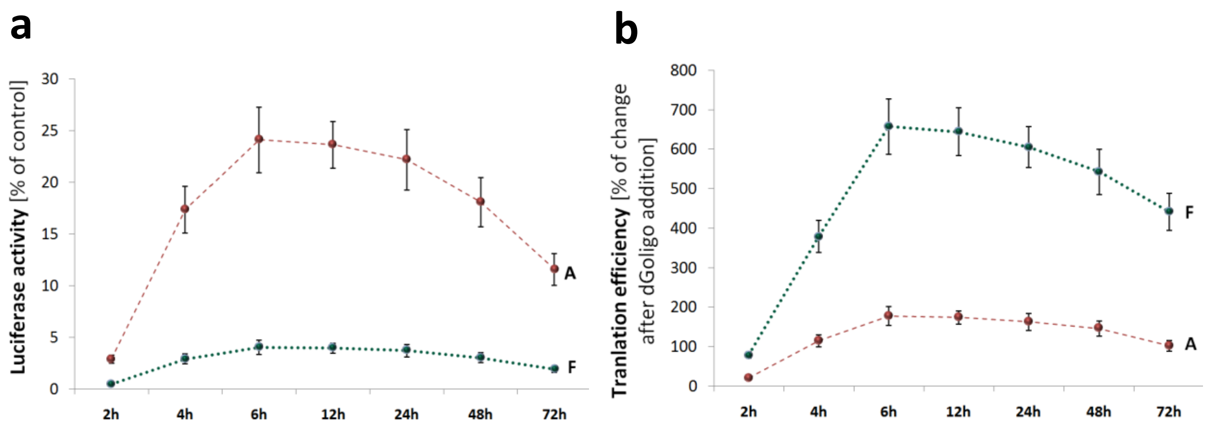

Fig S3. Time-course of protein synthesis rates in RTS 100 Wheat Germ CECF system.

(**a**) Effects of 5'UTR variants A (red line) and F (green line) on luciferase activities (reporter protein levels) after 2, 4, 6, 12, 24, 48 and 72 hours of coupled transcription-translation assay are shown relative to the control plasmid (Control). (**b**) Effects of both dG1 and dG4 (Table S3) on translation efficiency from pKS-A after 2, 4, 6, 12, 24, 48 and 72 hours (red line) or pKS-F (green line) are shown normalized to control (dG-). Experiments were performed in triplicate and shown as mean % luciferase activity ± SD. Data were analyzed by ANOVA, *p<0.001 vs. control.
