# Supplementary material for: A Novel Method for Gene-Specific Enhancement of Protein Translation by Targeting 5’UTRs of Selected Tumor Suppressors
Source: PLoS One. 2016 May 12;11(5):e0155359. doi: 10.1371/journal.pone.0155359 (PMC4865139; doi:10.1371/journal.pone.0155359)
Supplement: S4 Fig — (PDF) [file pone.0155359.s005.pdf]

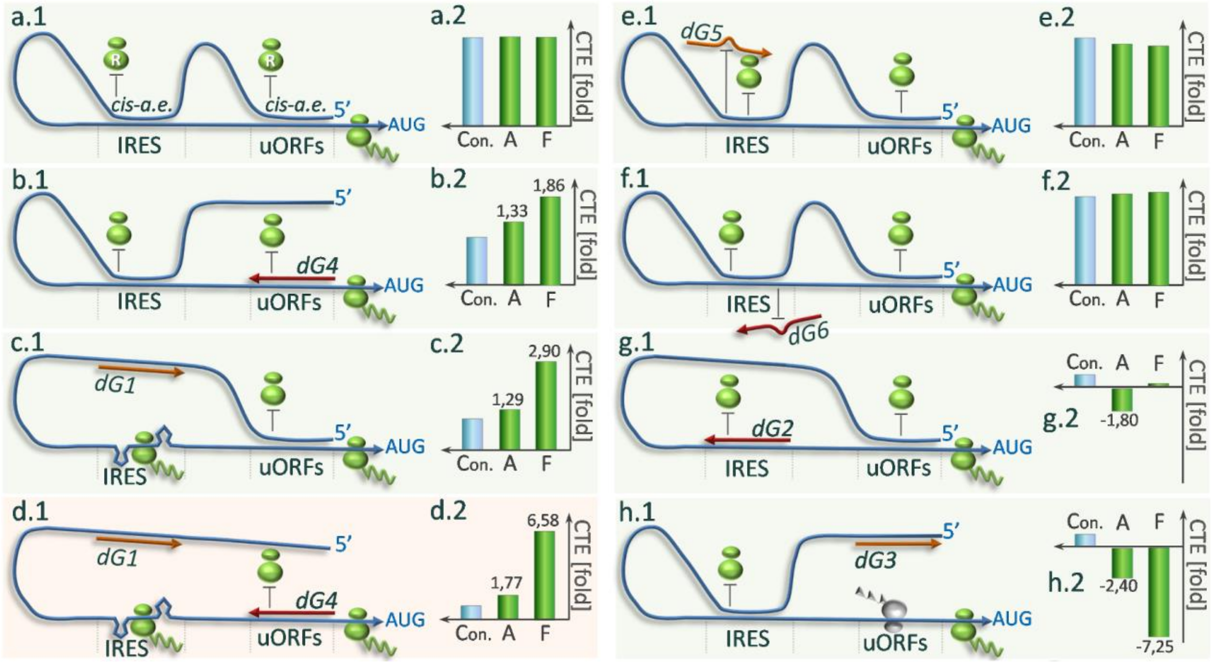

Fig S4. Proposed folding patterns of TRβ1 5'UTR after dG*oligo* supplementation.

Simplified models of secondary structures that could be linearized by dG*oligo*s (dGs) are shown (a.1-h.1) in relation to experimentally obtained data presenting changes in translation efficiency (CTE) after dG supplementation (a.2-h.2). Statistically significant CTEs ($p<0.001$) are indicated by fold of change in translation efficiency shown above or below green bars of 5'UTR variant A (A) and variant F (F) normalized to 100% of Control (Con. in blue) without supplementation of any dG. TRβ1 5'UTR is shown as blue curve ended by an arrow at AUG translation start codon. Two linked green ovals represent ribosome complex, that may be blocked by distant *cis*-acting element (*cis*-a.e) or *trans*-acting factor (*trans*-a.f., here dG). Putative Internal Ribosome Entry Site (IRES) involved in enhancement of cap-independent translation initiation (when free of distant *cis*-a.e.) and upstream Open Reading Frames (uORFs)–rich region, which may reduce translation initiation from the correct AUG start codon (when free of inhibitory *cis*-a.e. and *trans*-a.f.) are shown between doted vertical lines. (**a.1**) Theoretical state of naturally folded 5'UTR (without supplementation of any dG), with IRES and uORFs-rich domains are at least partially blocked by distant *cis*-acting elements resulting in basal translation level of correct protein. (**b.1**) Proposed model of dG4-mediated enhancement of translation efficiency, in which antisense dG4 can alter *Gibbs energy-dependent secondary structure formation* via direct binding to uORFs-rich region. This binding may block translation of truncated proteins originating from upstream AUGs, that finally may enhance translation initiation from correct AUG start codon. In the model, putative IRES domain stays at least partially blocked by distant *cis*-acting element. (**c.1**) Model of dG1-mediated enhancement of translation efficiency, where the sense dG1 can release e1 element containing putative IRES domain via binding to distant *cis*-acting sequences, normally interacting with the IRES sequence. This may allow for appropriate secondary structure formation of IRES domain needed for efficient cap-independent translation. In the model, uORFs-rich region stay at least partially blocked by naturally occurring distant *cis*-acting element of the 5'UTR, that finally may allow for translation initiation from correct AUG start codon. (**d.1**) Model of coupled action of dG1 and dG4 that mediate strong enhancement of translation efficiency (d.2). dG1 can release putative IRES domain via binding to distant *cis*-acting element and antisense dG4 can repress undesirable translation originated from uAUGs via direct binding to uORFs-rich region. This may allow for appropriate secondary structure formation of IRES domain needed for efficient cap-independent translation and blocking of uORFs-rich region required for efficient cap-dependent translation initiation from correct AUG start codon.

The observed enhancement of translation efficiency of folded variant F (d.2, F) may show that strongly folded 5'UTR variants have higher translational regulatory potential (TRP) when compared to weakly folded variants (d.2, A), which are efficiently translated even without addition of any *trans*-acting factors (here dGs). (**e.1**) Model of control dG5 action, that was blocked (e.2) by 3-nt insertion in the middle of the dG leading to insufficient similarity with 5'UTR mRNA sequence. (**f.1**) Model of control dG-6 (dG6) action, that was blocked (f.2) by 3-nt insertion mutation in the middle of the dG leading to insufficient complementarity with 5'UTR mRNA sequence and/or partial complementarity disturbing plant RNAi-related machinery, which could be involved in observed effects of completely complementary dG1 and dG4 (d.2). (**g.1**) Model of dG2 action leading to repression of translation efficiency (g.2 A) via direct binding of the dG to putative IRES domain. This may block cap-independent translation. uORFs-rich region stay at least partially blocked by naturally occurring distant *cis*-acting element of the 5'UTR that finally may allow for translation initiation from correct AUG start codon. (**h.1**) Model of dG3-mediated repression of translation, wherein sense dG3 can release uORFs-rich domain via binding to distant sequences, normally blocking the domain. This may allow for translation of truncated proteins originated from uAUGs reducing translation initiated from correct AUG start codon. In the model, putative IRES domain stay at least partially blocked by distant sequences inhibiting cap-independent translation initiation.
