# Supplementary material for: A Novel Method for Gene-Specific Enhancement of Protein Translation by Targeting 5’UTRs of Selected Tumor Suppressors
Source: PLoS One. 2016 May 12;11(5):e0155359. doi: 10.1371/journal.pone.0155359 (PMC4865139; doi:10.1371/journal.pone.0155359)
Supplement: S5 Fig — (PDF) [file pone.0155359.s006.pdf]

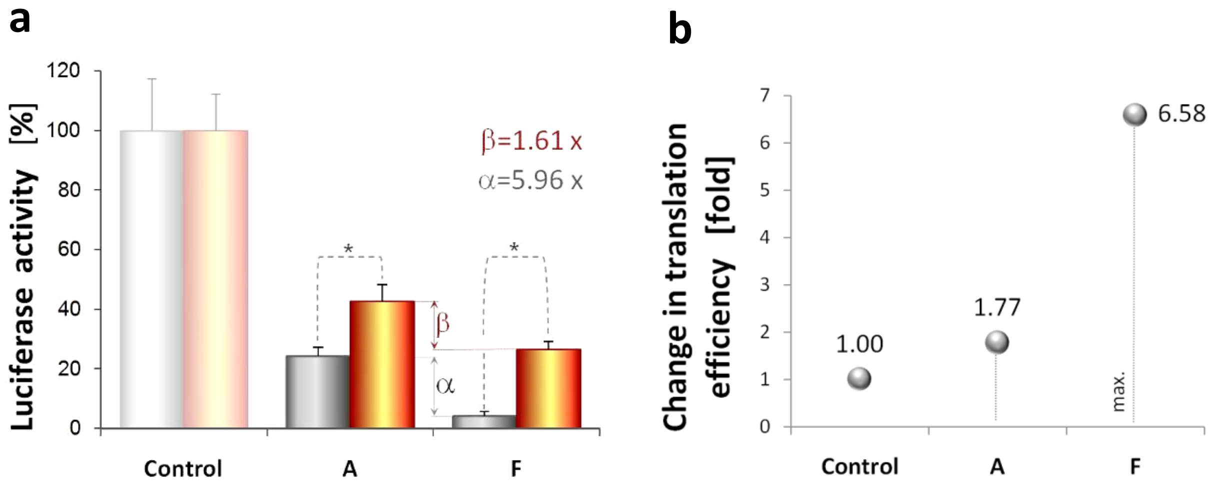

Fig S5. Change in translation efficiency after dG1 and dG4 supplementation.

(**a**) Effects of dG1 and 4 on translation efficiency (luciferase activity) of 5'UTR variants A and F are shown in orange bars, whereas basal translation rates of the 5'UTRs are indicated with grey bars; all results normalized to pKS-control plasmid (Control). α value, representing 5.96-fold higher basal translation rate of variant A compared to variant F, was reduced after treatment with dG1 and 4 to value β, showing only 1.61-fold higher translation of variant A when compared to variant F. α and β, which are indicated by bidirectional arrows, are experimentally obtained values of translational regulatory potential (TRP) of TRβ1 variants A and F that is in agreement with our predictions (Table-S2). After dG1+dG4 supplementation, strongly folded variant F exceeded the basal translation level of weakly folded variant A, showing that the strongly folded variant served as a translationally inactive /less-active transcript, which was recruited to translation through interaction with a *trans*-acting factor (here dG1 + dG4). Three independent experiments were performed in triplicate and shown as luciferase activity ± SD. Results obtained for variant A and F were analyzed by ANOVA followed by Dunnett's multiple comparison test; *p< 0.001 vs. basal translation rate was considered statistically significant. (**b**) Translation-enhancing effects of dG1 and dG4 on variants A and F that were normalized to control plasmid (Control) and shown as grey dots. Coupled action of dG1 and dG4 enhanced translation efficiency over 1.77- and 6.58-fold for the variant A and F respectively.
