# Supplementary material for: A Novel Method for Gene-Specific Enhancement of Protein Translation by Targeting 5’UTRs of Selected Tumor Suppressors
Source: PLoS One. 2016 May 12;11(5):e0155359. doi: 10.1371/journal.pone.0155359 (PMC4865139; doi:10.1371/journal.pone.0155359)
Supplement: S6 Fig — (PDF) [file pone.0155359.s007.pdf]

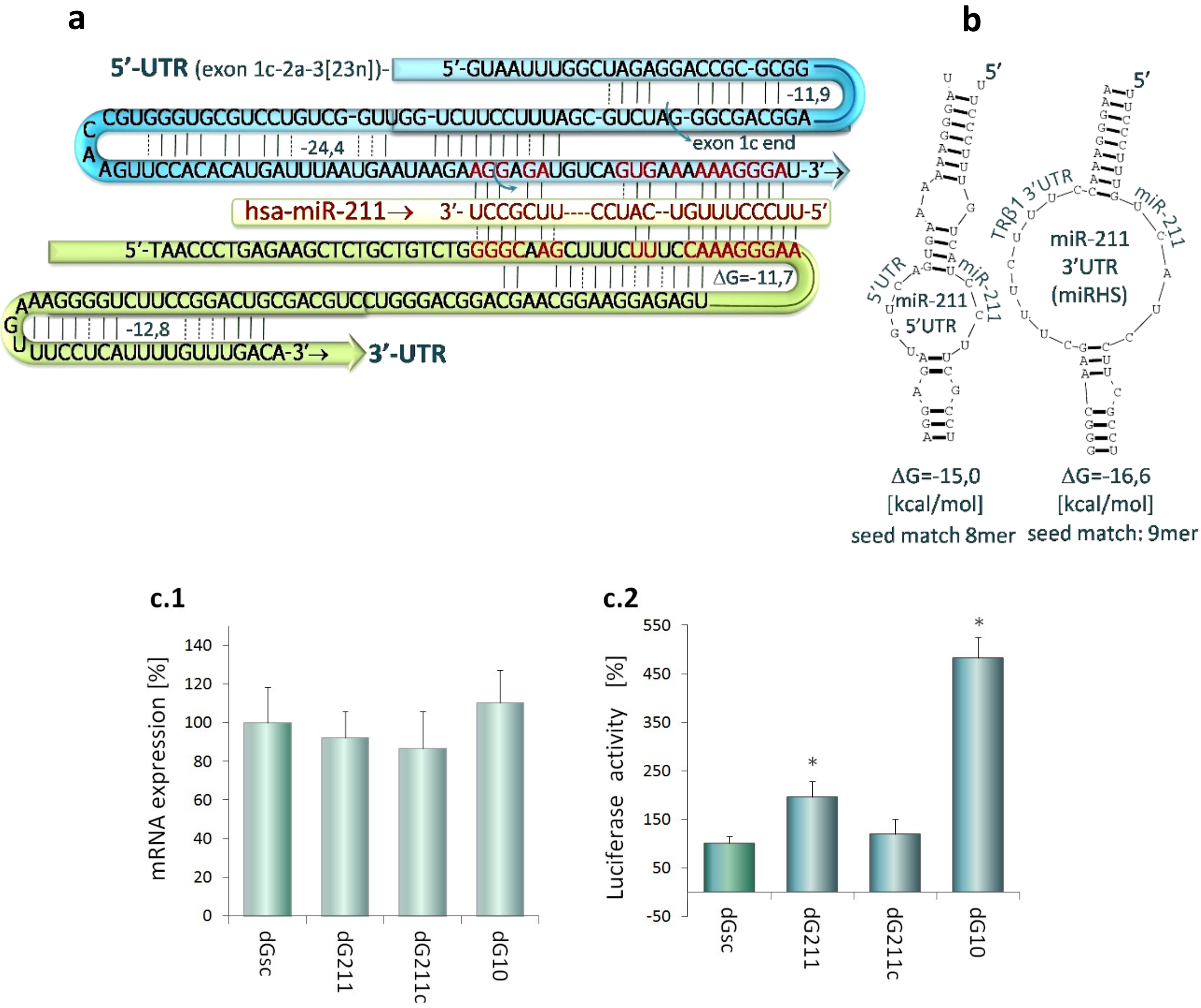

Fig S6. Potential hsa-miR-211 target sites within TRβ1 3'UTR and 5'UTR.

(**a**) Target sites for a microRNA - hsa-miR-211 (miRBase Acc. no. MI0000287) in TRβ1 untranslated regions are highlighted in blue (5'UTR) and green (3'UTR). This non-selective microRNA binding may influence secondary structures of both UTRs and contribute to changes in Gibbs energy that finally may affect protein synthesis. (**b**) Target sites within TRβ1 UTR sequences were identified using miRBase (http://www.mirbase.org) and RNAhybrid (http://bibiserv.techfak.uni-bielefeld.de/rnahybrid/) that uses a Gibbs energy (ΔG) algorithm to calculate favorable binding interactions between a microRNA and potential target sites within mRNA. (**c**) Effects of 2'-O-methyl RNA modified hsa-miR-211 termed dG*oligo*-hsa-miR211 (dG211, Table S3), dG*oligo*-hsa-miR211-3p (dG211c, ~complementary to dG211), dG10 and scrambled control (dGsc) on luciferase transcription (**c.1**) and translation (**c.2**) in Caki-2 cells transfected with pGL3-A (see Appendix S1), containing TRβ1 5'UTR variant A, luciferase coding sequence and irrelevant 3'UTR. miRBase and RNAhybrid - based analysis revealed no hsa-miR-211 and hsa-miR211-3p targets within pGL3-A 3'UTR, suggesting that the observed effects (c) could be mediated through TRβ1 5'UTR. Although our dG10 (designed on the basis of TRβ1 5'UTR) showed the strongest translation-enhancing effect in Caki-2 cells (see Fig 6), hsa-miR-211 (dG211) enhanced translation by 1.95-fold as well and had no effects on luciferase mRNA levels. Results from three independent experiments performed in triplicates are shown as mean % mRNA (a) or luciferase activity (b) ± SD. Data analyzed by ANOVA followed by Dunnett's multiple comparison test. *p<0.001 vs. control.
