# Supplementary material for: A Novel Method for Gene-Specific Enhancement of Protein Translation by Targeting 5’UTRs of Selected Tumor Suppressors
Source: PLoS One. 2016 May 12;11(5):e0155359. doi: 10.1371/journal.pone.0155359 (PMC4865139; doi:10.1371/journal.pone.0155359)
Supplement: S7 Fig — (PDF) [file pone.0155359.s008.pdf]

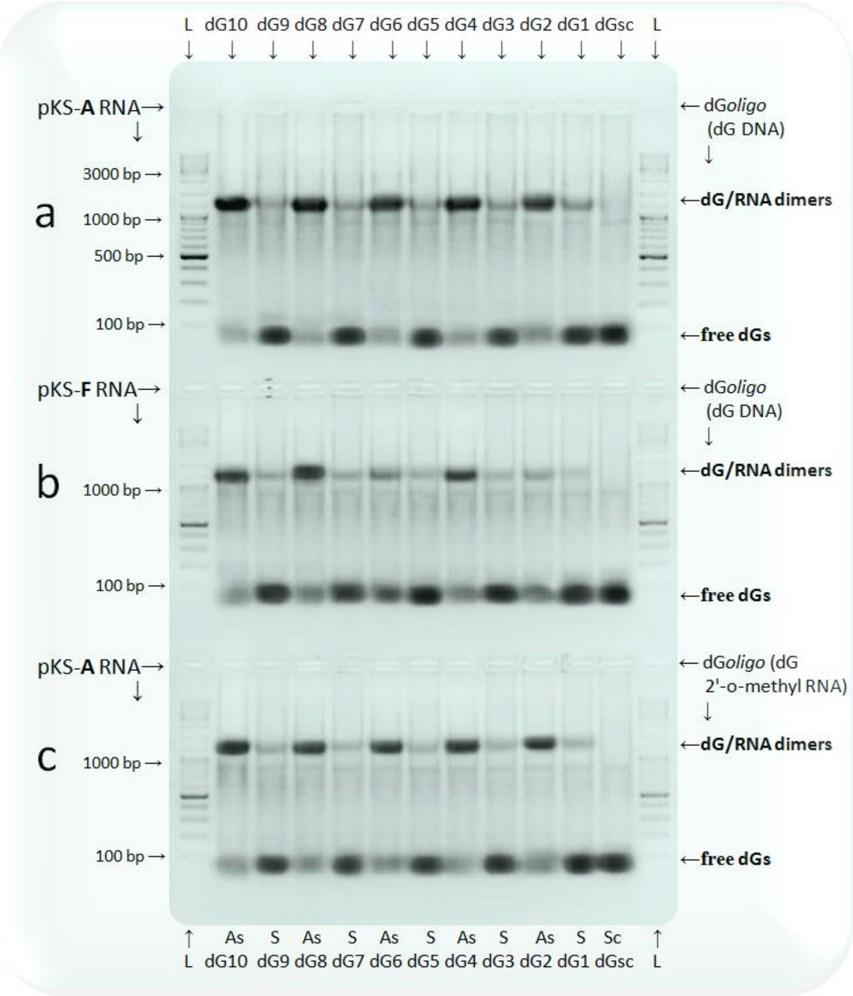

Fig S7. dG*oligo* binding capacity.

SYBR Green I stained agarose gel electrophoretograms show bands formed by a hybridized dG*oligo* (dG) and a target RNA obtained by *in vitro* T7 polymerase-mediated transcription of pKS-A or pKS-F plasmids (see Appendix S1). (**a**) Binding of DNA-based dGs to the pKS-A RNA. (**b**) Binding of DNA-based dGs to the pKS-F RNA. (**c**) Binding of 2'-O-methyl modified RNA dGs to the pKS-A RNA. Arrowheads on the right of each panel indicate positions of dG*s*/RNAs dimmers (between 1000-3000bp) and free dGs (<100bp). Due to lower binding capacity of Sybr Green I to free RNA, it can be only slightly seen below the dG*s*/RNAs pairs. All dGs, which are shown here individually, were designed originally as antisenses (dG2, dG4, dG6, dG8, dG10) directly recognizing regulatory sequence within TRβ1 5'UTR b) senses (dG1, dG3, dG5, dG7, dG9) that could release homologous region by binding to a distant sequence folding within this region (see Table S3, Fig S1). Antisense-like dGs (As) generate stronger band signals when compared to sense (S) dGs, which may share only partial complementarity with the distant 5'UTR sequence fragments. Although sense-like dGs exerted weak binding capacity, their translation-enhancing action could be released by partial complementarity with the distant 5'UTR sequences unfolding the homologous sequences or via interaction with other trans-acting factors. Scrambled control (dGsc) with an irrelevant (random) sequence revealed no interaction with the RNA, thus, confirming the specificity of binding by other dGs. Due to different exon 1e/2a boundary of variant F compared to variant A (ex1c/2a), only half of dG2 and dG6 shares sequence with 5'UTR variant F (pKS-F) that results in a weak binding capacity (electrophoretogram b). GeneRuler DNA Ladder Mix (Thermo Scientific) served as a marker ladder (L), shown on the right and left of each gel. The observed binding capacity and selectivity of dGs was also tested independently, by dG-primed reverse transcription (Fig S8).
