# Supplementary material for: A Novel Method for Gene-Specific Enhancement of Protein Translation by Targeting 5’UTRs of Selected Tumor Suppressors
Source: PLoS One. 2016 May 12;11(5):e0155359. doi: 10.1371/journal.pone.0155359 (PMC4865139; doi:10.1371/journal.pone.0155359)
Supplement: S8 Fig — (PDF) [file pone.0155359.s009.pdf]

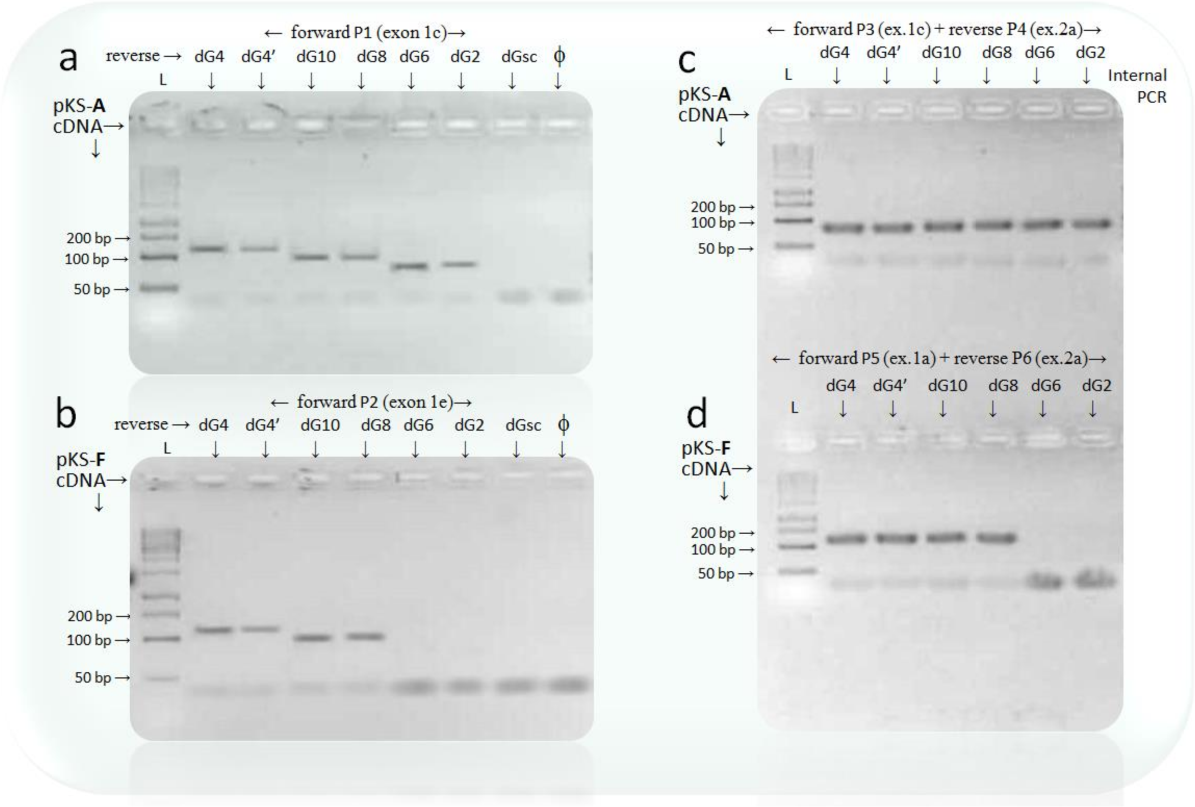

Fig S8. Binding selectivity confirmed by dG*oligo*-primed reverse transcription.

Here we show PCR products obtained with dG*oligos* (dGs) used to drive synthesis of specific cDNAs in reaction of reverse transcription. This approach was based on primer extension by reverse transcriptase, which requires complementarity between a target sequence and, at least, 3'-end of an oligonucleotide (see Appendix S1). Panels a, b, c and d present selected SYBR Green I stained agarose gel electrophoretograms showing PCR products obtained on the basis of cDNA matrixes that were synthesized using antisense-like dG*s* including dG2, dG4, dG4' (dG4 from control synthesis), dG6, dG8, dG10 and control dGsc (scrambled). (**a**) PCR-amplified DNA fragments obtained on the basis of pKS-A cDNA, synthesized using one of the mentioned antisense-like dGs or dGsc. Common forward primer recognizing 5' end of exon 1c (P1) and one of the dGs (dG2, 4, 6, 8, 10) as a reverse primer were used in the PCR. (**b**) PCR fragments obtained on the basis of pKS-F cDNA, synthesized before using one of dGs or dGsc. Common forward primer recognizing 5' end of exon 1e (P2) and one of the dGs as a reverse primer were used. (**c**) Internal PCR fragments obtained on the basis of pKS-A cDNA, synthesized before using one of the dGs or dGsc. Common forward (P3) and reverse (P4) primer recognizing 3' end of exon 1c and exon 2a were used. (**d**) PCR fragments obtained on the basis of pKS-F cDNA, synthesized before using one of the dGs or dGsc. Common forward (P5) and reverse (P6) primer recognizing 3' end of exon 1a and exon 2a were used. ϕ indicates a control PCR-sample containing H_2O instead of the cDNA. Arrowheads on the left of each panel indicate size of bands of marker ladder (L). Except for dGsc, all tested dGs mediated reverse transcription showing their binding selectivity (a, b, c, d). However, due to different exon 1e/2a boundaries of variant F compared to variant A (exon 1c/2a), only half of dG2 and dG6 shares sequence with 5'UTR variant F (pKS-F). In case of 5'UTR variant F, we observed lack of PCR products primed by dG2 and dG6 (b, d), confirming that 3'-end of these dGs do not form non-specific base-pairs with their targets.
