# Supplementary material for: A Novel Method for Gene-Specific Enhancement of Protein Translation by Targeting 5’UTRs of Selected Tumor Suppressors
Source: PLoS One. 2016 May 12;11(5):e0155359. doi: 10.1371/journal.pone.0155359 (PMC4865139; doi:10.1371/journal.pone.0155359)
Supplement: S9 Fig — (PDF) [file pone.0155359.s010.pdf]

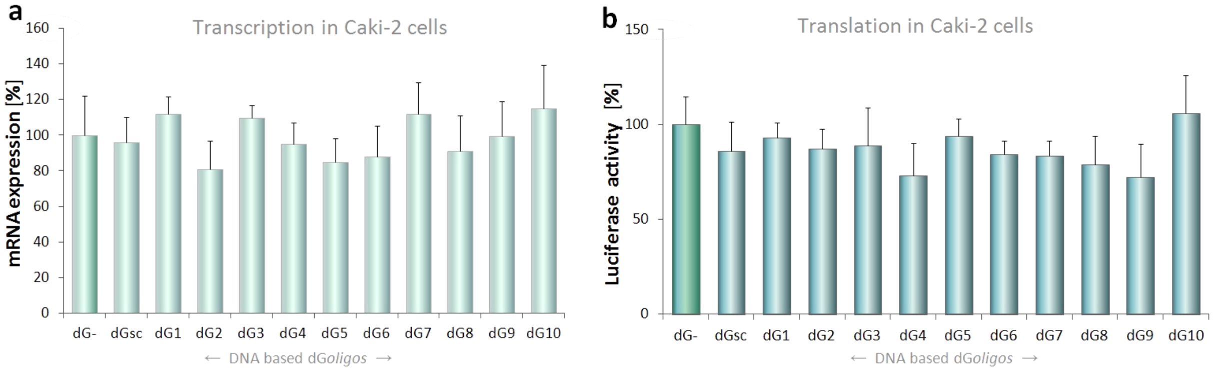

Fig S9. DNA-based dG*oligo*-mediated effects in Caki-2 cells.

Here we show that DNA dG*oligos* (dGs) have no significant effects on luciferase transcription (**a**) and translation (**b**) in Caki-2 cells transfected with pGL3-A. These data are consistent with previously reported findings that unmodified deoxyoligonucleotides can be rapidly degraded by nucleases and are of limited utility in mammalian cells (see results in the article). Results from three independent experiments performed in triplicates are shown as mean % mRNA (a) or luciferase activity (b) ± SD. Data analyzed by ANOVA followed by Dunnett's multiple comparison test. *$p<0.001$ vs. control.
