# Supplementary material for: A Novel Method for Gene-Specific Enhancement of Protein Translation by Targeting 5’UTRs of Selected Tumor Suppressors
Source: PLoS One. 2016 May 12;11(5):e0155359. doi: 10.1371/journal.pone.0155359 (PMC4865139; doi:10.1371/journal.pone.0155359)
Supplement: S1 Table — (PDF) [file pone.0155359.s011.pdf]

Supporting tables

5'UTR variant*	GenBank Acc. No.:	Length [nt]	uAUGs [no.]	GC-content [%]	ΔG [kcal/mol]	Translation** efficiency [%]
control	-	29	0	55.17	-6.3	100
**A**	AY286465.1	211	4	54.98	**-69.0**	24.09
B	AY286466.1	229	5	54.15	-82.0	12.08
C	AY286467.1	195	4	56.41	-77.5	14.99
D	AY286468.1	314	5	48.09	-95.0	7.01
E	AY286469.1	305	5	47.54	-92.5	7.99
**F**	AY286470.1	339	7	58.41	**-128.9**	4.02
G	AY286471.1	388	6	52.84	-127.0	3.00

Table S1. Basic characteristics of selected TRβ1 5’UTR variants A-G.

Prediction of secondary structures of 5’UTRs was performed as described in bioinformatic analysis. The least and most folded TRβ1 variants are shown in bold. uAUGs represent start codons located upstream of the main start codon and Kozak consensus sequence. *TRβ1 5’UTR splice variants termed according to Frankton et al 2004. ** Translation efficiency measured with RTS 100 Wheat Germ CECF system.
