# Supplementary material for: A Novel Method for Gene-Specific Enhancement of Protein Translation by Targeting 5’UTRs of Selected Tumor Suppressors
Source: PLoS One. 2016 May 12;11(5):e0155359. doi: 10.1371/journal.pone.0155359 (PMC4865139; doi:10.1371/journal.pone.0155359)
Supplement: S2 Table — (PDF) [file pone.0155359.s012.pdf]

Column →	2	3	4	5	6	7	8	9
[&]**Abbreviation →**	Acc. No.	ΔG [kcal/mol]	ETE [%]	**TE** [%]	ΔG [kcal/mol]	**TE** [%]	ΔG shift [%]	TTE shift [%]
**Sequence →**	reference	reference	reference	reference	*substituted*	*substituted*	substituted/ reference	substituted/ reference
**Value →**		theoretical	experimental	theoretical	theoretical	theoretical	theoretical	theoretical
**Equation →**		determined using RNA-structure prog.	determined with translation assay	=127.24* e^(0.0284* column-3)	determined using RNA-structure prog.	=127.24* e^(0.0284* column-6)	=|100%- column6/3 *100%|	=|100%- column7/5 *100%|
Reference 5'UTR variant ↓	GenBank Acc. No. – reference variants	ΔG of reference variants	Experimental results of translation efficiency (TE)	Predicted TE of reference variants	ΔG of substituted variant	Predicted TE of substituted variants	Shift in Gibbs energy (between substituted and reference variants)	Shift in predicted theoretical TE (between substituted and reference variants)
Control	-	-6.3	100	106.44	-6.3	106.44	0.00	0.00
**A**	AY286465.1	-69.0	24.09	17.94	-67.5	18.72	**2.17**	**4.35**
B	AY286466.1	-82.0	12.08	12.40	-78.8	13.58	3.90	9.51
C	AY286467.1	-77.5	14.99	14.09	-75.2	15.04	2.97	6.75
D	AY286468.1	-95.0	7.01	8.57	-90.3	9.80	**4.95**	14.28
E	AY286469.1	-92.5	7.99	9.20	-88.4	10.34	4.43	12.35
**F**	AY286470.1	-128.9	4.02	3.27	-123.0	3.87	4.58	**18.24**
G	AY286471.1	-127.0	3.00	3.45	-121.4	4.05	4.41	17.24

Table S2. Prediction of translational regulatory potential of 5'UTRs.

This analysis was performed to determine translation regulatory potential (TRP) of various TRβ1 5'UTRs and was used in dG*enhancer* calculations (see Appendix S1). The TRP predictions are shown as numerical parameters such as shifts in *theoretical translation efficiency* (TTE shifts, column-9) and Gibbs energy (ΔG shifts, column-8). The TTE shifts were calculated by dividing predicted TTE of virtually *substituted* TRβ1 5'UTR variants (column-7) and reference non-*substituted* variants (column-5). The Gibbs energy shifts were calculated by dividing Gibbs energy values of the *substituted* (column-6) and reference variants (column-3). Computational prediction of the translation efficiency (TE) was performed on the basis of exponential trend-line equation correlating experimentally obtained values of translation efficiency and the Gibbs energies of the 5'UTRs ($y=127.24 \cdot e^{0.0284 \cdot x}$, where x means calculated Gibbs energy, number e - constant = 2.718, y - translation efficiency value). Extreme values (min., max.) of the shift in Gibbs energy and TTE shifts are shown in bold. *Substituted* variant D and F were predicted (by ΔG shifts and TTE shifts, respectively) to have the highest translational regulatory potential. In contrast to ΔG shifts, TTE shifts include calculations from a trend-line equation correlating experimental results of translation efficiency with 5'UTR Gibbs energies. In our experimentally obtained data variant F was found to have the highest dG-triggered TRP (Fig 4.d, Fig S5), whereas variant D has been previously reported to drive efficient luciferase expression in kidney-derived COS-7 cells (Frankton et al. 2004) that is in concordance with the prediction in this table. These data may show that 5'UTR TRP should always be estimated in the context of a translation system involving additional parameters of translation machinery such as various *trans*-acting factors that, besides the Gibbs energy, may influence protein synthesis efficiency.

[&]**Abbreviations**: TRP – Translational Regulatory Potential; ETE – Experimentally determined Translation Efficiency; TTE – Theoretical Translation Efficiency, calculated on the basis of trend-line equation (see below) and values of ΔG – Gibbs Energy; Acc. No. – Accession Number of Gene Bank (NCBI); reference sequence – correct sequence; substituted sequence – containing Small Nucleotide Polymorphism (SNP); shift - change between calculated values (ΔG [kcal/mol] or translation efficiency [%]) of reference and substituted 5'UTR variant.
