# Supplementary material for: A Novel Method for Gene-Specific Enhancement of Protein Translation by Targeting 5’UTRs of Selected Tumor Suppressors
Source: PLoS One. 2016 May 12;11(5):e0155359. doi: 10.1371/journal.pone.0155359 (PMC4865139; doi:10.1371/journal.pone.0155359)
Supplement: S3 Table — (PDF) [file pone.0155359.s013.pdf]

dG*oligo* full name and target site within 5’UTR	Short -cut	Position 5'→3' [nt]	Target sequence (element)	Structure / strand orientation	Design method	dG*oligo* Sequence 5'→3'
dG*oligo*1-TRβ1vA-IRES-ex1c/ex2a-F	dG1	86→108	e1 TRβ1	sense	*manual*	GGAGGCAGCGGGATCTGCGATTT
dG*oligo*2-TRβ1vA-anti-IRES-ex1c/ex2a-R	dG2	108→86	e1 TRβ1	antisense	*manual*	AAATCGCAGATCCCGCTGCCTCC
dG*oligo*3-TRβ1vA-uAUGs-ex3/ex2a-F	dG3	159→179	e3 TRβ1	sense	*manual*	TGAATAAGAAGGAGATGTCAG
dG*oligo*4-TRβ1vA-anti-uAUGs-ex3/ex2a-R	dG4	179→159	e3 TRβ1	antisense	*manual*	CTGACATCTCCTTCTTATTCA
dG*oligo*5-TRβ1vA-mirSenseIres- ex1c/ex2a-F	dG5	86→108	e1 TRβ1	miRNA-like sense control	*manual*	GGAGGCAGCGGGtagATCTGCGATTT
dG*oligo*6-TRβ1vA-anti-mirAntiSenseIres- ex1c/ex2a-R	dG6	108→86	e1 TRβ1	miRNA-like antisense control	*manual*	AAATCGCAGATctaCCCGCTGCCTCC
dG*oligo*7-TRβ1vA-cisAE-ex2a-F	dG7	129→151	e2 TRβ1	sense	automated dG*enhancer*	CGTGGGTGCCAAGTTCCACACAT
dG*oligo*8-TRβ1vA-anti-cisAE-ex2a-R	dG8	151→129	e2 TRβ1	antisense	automated	ATGTGTGGAACTTGGCACCCACG
dG*oligo*9-TRβ1vA-mirSenseCisAE-ex2a-F	dG9	129→151	e2 TRβ1	miRNA-like sense	automated dG*enhancer*	CGTGGGTGCCAActaGTTCCACACAT
dG*oligo*10-TRβ1vA-mirAntisense-cisAE-ex2a-R	dG10	151→129	e2 TRβ1	miRNA-like antisense	automated	ATGTGTGGAACTctcTGGCACCCACG
dG*oligo*Sc-TRβ1vA	dGsc	108→86	-	nonsense / scrambled control	automated GeneScript	GCCATCACTACGCCTACCTCAGTCGA
dG*oligo*1p16-p16INK4a5'UTR-sense-U	dG1p16	280→301	e1 p16	sense	*manual* and automated dG*enhancer*	AGCAGGCAGCGGGCGGCGGGGA
dG*oligo*2p16- p16INK4a5'UTR-antisense-L	dG2p16	301→280	e1 p16	antisense	*manual* and automated dG*enhancer*	TCCCCGCCGCCCGCTGCCTGCT
dG*oligo*3p16-p16INK4a5'UTR-miRNA-U	dG3p16	280→301	e1 p16	miRNA-like sense	automated dG*enhancer*	AGCAGGCAGCGGtaGCGGCGGGGA
dG*oligo*G4p16-p16INK4a5'UTR-miRNA-L	dG4p16	301→280	e1 p16	miRNA-like antisense	automated dG*enhancer*	TCCCCGCCGCtaCCGCTGCCTGCT
dG*oligo*5p16-p16INK4a5'UTR-sense-U	dG5p16	2→22	e2 p16	sense	automated dG*enhancer*	GAGGGCTGCTTCCGGCTGGTG
dG*oligo*6p16- p16INK4a5'UTR-antisense-L	dG6p16	22→2	e2 p16	antisense	automated dG*enhancer*	CACCAGCCGGAAGCAGCCCTC
dG*oligo*Gscp16-p16INK4a5'UTR	dGsc p16	301→280	-	nonsense / scrambled control	automated GeneScript	GTCCTCGCCTCCGCACCGGTCCTC
dG*oligo*-hsa-miR211	dG211	192→168	e3/e4 TRβ1	miRNA	miRBase	TTCCCTTTGTCATCCTTCGCCT
dG*oligo*-hsa-miR-211-3p	dG211c	168→192	e3/e4 TRβ1	Complem. miRNA	miRBase	GCAGGGACAGCAAAGGGGTGC

Table S3. List of dG*oligos* (dGs) used in the study.

Position in 5’UTR indicates dG recognition site in 5’UTR variant A of TRβ1 (TRβ1vA, GeneBank acc. no.: AY286465.1) encoded by *THRB* gene and in p16INK4a 5'UTR (NM_000077.4) encoded by *CDKN2A*. 2-3-nucleotide, microRNA-mimicking loop is indicated with lowercase letters, dGsc-scrambled control.

dG*enhancer* - see Appendix S1
GeneScript - https://www.genscript.com/ssl-bin/app/scramble
miRBase - http://www.mirbase.org/
