# Supplementary material for: A Novel Method for Gene-Specific Enhancement of Protein Translation by Targeting 5’UTRs of Selected Tumor Suppressors
Source: PLoS One. 2016 May 12;11(5):e0155359. doi: 10.1371/journal.pone.0155359 (PMC4865139; doi:10.1371/journal.pone.0155359)
Supplement: S4 Table — (PDF) [file pone.0155359.s014.pdf]

Oligonucleotide name	Oligonucleotide Sequence 5′ → 3′	Recognition site /target	Description	Strand orientation
T7prom-f	TATGTAATACGACTCACTATAGGG	T7 promoter, only DNA	Real Time PCR, PCR product B	sense, forward
Luc-RT-f	CACCATGGAAGACGCCAA	Luciferase Kozak cons. seq./coding sequence	Real Time PCR, PCR product A	sense, forward
Luc-RT-r	CCAGCGGTTCCATCTTCCAG	Luciferase sequence	Real Time PCR, PCR product A	antisense, reverse
Luc-rev-r	GATGTCCACCTCGATATGTGC	Luciferase sequence	Reverse Transcription	antisense, reverse
Ffir	ATCGTGGACCGCCTGAAGTC	Firefly luciferase	Real-Time-PCR	forward
Rfir	ACGACGGCGGCAGGCAGC	Firefly luciferase	Real-Time-PCR	reverse
Fren	TGAGGAGTTCGCTGCCTACC	*Renilla* luciferase	Real-Time-PCR	forward
Ren	TGCGGACAATCTGGACGACG	*Renilla* luciferase	Real-Time-PCR	reverse
P1	GTAATTTGGCTAGAGGACC	5′ end of TRβ1 ex. 1c (specific to variant A)	PCR	forward
P2	GTCCTAGAAAGGAAAGCACAG	5′ end of TRβ1 ex. 1e (specific to variant F)	PCR	forward
P3	AGGACCGCGCGGAGGCAG	3′ end of TRβ1 ex.1c (specific to variant A)	PCR	forward
P4	TCGAAGCTTCAGTCAGTGG-CAACCAGAAGGAAATCGCAGAT	5′end of TRβ1 2a with 5′overhang	PCR	reverse
P5	TGACATTTTGCAGGACTCG	3′end of TRβ1 ex. 1a (specific to variant F)	PCR	forward
P6	CCAACCAGAAGGAAATCGCAG	TRβ1 2a	PCR	reverse
S0.F	CGAGGGCTGCTTCCGGCT	p16INK4a 5'UTR	PCR0	forward
S0.R	GCTGCTCCCCGCTGCCCGCT	p16INK4a 5'UTR	PCR0	reverse
SI.F (cSIII.F – T7p. – p16 5′UTR)	TCGAAGCTTCAGTCAGT-ATATGTAATACGACTCACTATAGGG-T-CGAGGGCTGCTTCCGGCT	p16INK4a 5'UTR linear construct	PCRI	forward
SI.R(cSII.F)	GTTTTTGGCGTCTTCCAT-GCTGCTCCCCGCCGCCCGCT	p16INK4a 5'UTR linear construct	PCRI	reverse
SII.F	TGGAAGACGCCAAAAACAT	p16INK4a 5'UTR linear construct	PCRII	forward
SII.R(cSIII.R)	TGACCCTGGTTGACCCTACT-CCGGAAGGAGCTGACTGG	p16INK4a 5'UTR linear construct	PCRII	reverse
SIII.F	TCGAAGCTTCAGTCAGT	p16INK4a 5'UTR linear construct	PCRIII	forward
SIII.R	TGACCCTGGTTGACCCTACT	p16INK4a 5'UTR linear construct	PCRIII	reverse

Table S4. List of primers used in Real-Time and classic PCR.

This table contains sequences of DNA oligonucleotides (primers) used in reverse transcription and PCR assays of TRβ1 (*THRB*) and p16INK4a (*CDKN2A*; see Appendix S1).
